# The multispecies microbial cluster of *Fusobacterium*, *Parvimonas*, *Bacteroides* and *Faecalibacterium* as a precision biomarker for colorectal cancer diagnosis

**DOI:** 10.1002/1878-0261.13604

**Published:** 2024-02-17

**Authors:** Kelly Conde‐Pérez, Pablo Aja‐Macaya, Elena Buetas, Noelia Trigo‐Tasende, Mohammed Nasser‐Ali, Soraya Rumbo‐Feal, Paula Nión, Elsa Martín‐De Arribas, Lara S. Estévez, Begoña Otero‐Alén, José F. Noguera, Ángel Concha, Simón Pardiñas‐López, Miguel Carda‐Diéguez, Igor Gómez‐Randulfe, Nieves Martínez‐Lago, Susana Ladra, Luis M. A. Aparicio, Germán Bou, Álex Mira, Juan A. Vallejo, Margarita Poza

**Affiliations:** ^1^ Microbiome and Health Group (meiGAbiome), Microbiology Research Group, Institute of Biomedical Research (INIBIC) – Interdisciplinary Center for Chemistry and Biology (CICA) – University of A Coruña (UDC) – CIBER de Enfermedades Infecciosas (CIBERINFEC‐ISCIII), Servicio de Microbiología University Hospital of A Coruña (CHUAC) A Coruña Spain; ^2^ Genomic and Health Department, FISABIO Foundation Center for Advanced Research in Public Health Valencia Spain; ^3^ Database Laboratory, Research Center for Information and Communication Technologies (CITIC) University of A Coruña (UDC) A Coruña Spain; ^4^ Pathology Service and Biobank University Hospital of A Coruña (CHUAC) A Coruña Spain; ^5^ Surgery Service University Hospital of A Coruña (CHUAC) A Coruña Spain; ^6^ Periodontology and Oral Surgery, Pardiñas Medical Dental Clinic – Cell Therapy and Regenerative Medicine Group Institute of Biomedical Research (INIBIC) A Coruña Spain; ^7^ Medical Oncology Department University Hospital of A Coruña (CHUAC) A Coruña Spain; ^8^ Microbiome and Health Group, Faculty of Sciences University of A Coruña (UDC) A Coruña Spain

**Keywords:** bacterial consortium, biomarkers, colorectal cancer, microbiome, oral‐gut axis, periodontal disease

## Abstract

The incidence of colorectal cancer (CRC) has increased worldwide, and early diagnosis is crucial to reduce mortality rates. Therefore, new noninvasive biomarkers for CRC are required. Recent studies have revealed an imbalance in the oral and gut microbiomes of patients with CRC, as well as impaired gut vascular barrier function. In the present study, the microbiomes of saliva, crevicular fluid, feces, and non‐neoplastic and tumor intestinal tissue samples of 93 CRC patients and 30 healthy individuals without digestive disorders (non‐CRC) were analyzed by 16S rRNA metabarcoding procedures. The data revealed that *Parvimonas*, *Fusobacterium*, and *Bacteroides fragilis* were significantly over‐represented in stool samples of CRC patients, whereas *Faecalibacterium* and *Blautia* were significantly over‐abundant in the non‐CRC group. Moreover, the tumor samples were enriched in well‐known periodontal anaerobes, including *Fusobacterium*, *Parvimonas*, *Peptostreptococcus*, *Porphyromonas*, and *Prevotella*. Co‐occurrence patterns of these oral microorganisms were observed in the subgingival pocket and in the tumor tissues of CRC patients, where they also correlated with other gut microbes, such as *Hungatella*. This study provides new evidence that oral pathobionts, normally located in subgingival pockets, can migrate to the colon and probably aggregate with aerobic bacteria, forming synergistic consortia. Furthermore, we suggest that the group composed of *Fusobacterium*, *Parvimonas*, *Bacteroides*, and *Faecalibacterium* could be used to design an excellent noninvasive fecal test for the early diagnosis of CRC. The combination of these four genera would significantly improve the reliability of a discriminatory test with respect to others that use a single species as a unique CRC biomarker.

## Graphical abstract

Periodontal pathogens showed the same co‐occurrence patterns in the subgingival environment and in the tumors of colorectal cancer (CRC) patients. Oral pathobionts can migrate to the gut by forming synergistic consortia. Moreover, the group composed of *Fusobacterium*, *Parvimonas*, *Bacteroides*, and *Faecalibacterium* could be used to design a noninvasive fecal test for CRC diagnosis, improving the reliability of other discriminatory tests.

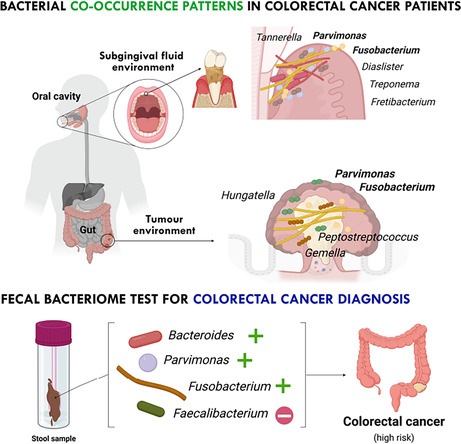

**Abbreviations**
16S rRNA: 16S ribosomal RNA gene
Ac: adenocarcinoma samples
AECC: Spanish Association Against Cancer
AEMPS: Spanish Agency for Medicines and Healthcare Products
ASV: amplicon sequence variant
AUC: area under the curve
BFT: *Bacteroides fragilis* toxin
CBCT: cone beam computed tomography
CCA: canonical correlation analysis
CHUAC: University Hospital of A Coruña
CMS1: consensus molecular subtype 1
CRC: colorectal cancer
DAA: differential abundance analysis
DMFT: decayed, missing, and filled teeth
DMM: dirichlet multinomial mixtures
F: feces samples
FOBT: fecal occult blood tests
GCF: gingival crevicular fluid
GIT: gastrointestinal tract
ISCIII: Carlos III Health Institute
LOOCV: leave‐one‐out cross‐validation
NGS: next‐generation sequencing
NM: non‐neoplastic colon tissue samples
NMDS: non‐metric multidimensional scaling
non‐CRC: healthy individuals without digestive disorders
PD: periodontal disease
RA: relative abundance
ROC: receiver operating characteristic
S: saliva samples
SCFA: short‐chain fatty acids
SERGAS: Galician Health Service

## 1 Introduction

Colorectal cancer (CRC) is one of the most common types of cancer worldwide, after breast, prostate, and lung cancers (considering both sexes and all ages). Nevertheless, it is the second type of cancer that causes the most deaths worldwide, with 935 173 deaths in 2020 [1]. In Spain, CRC is a malignant neoplasm with the highest incidence [2]; specifically, the province of A Coruña (Galicia, NW of Spain) is an area where the incidence and mortality of CRC are increasing every year [2]. The usual precursors of CRC are colorectal polyps and the progression from benign polyps to carcinomas, which occurs very slowly and sporadically, usually taking approximately 10–15 years if there is no specific genetic syndrome (which occurs only in ~ 5% of cases) [3]. Most common symptoms of CRC are unspecific (e.g., presence of mucus or blood in stool, abdominal or pelvic pain), and most of the time patients do not show any signs until illness worsens, so colorectal tumors are frequently diagnosed at advanced stages [4, 5, 6]. Later CRC detection decreases the survival rate and increases the morbidity of patients, with a 5‐year survival rate of 75–90% if cancer is detected in early stages (e.g., stages I–II) and only ~ 15% when CRC is diagnosed in more serious and advanced stages (e.g., stage IV) [6]. Stool tests are easy and inexpensive methods for selecting CRC high‐risk individuals. In Spain, with the aim of increasing the detection of cases, CRC follow‐up programs have been implemented for individuals aged 50 years and above. These programs utilize fecal occult blood tests (FOBT), which promote a downward trend in CRC mortality [7]. Nevertheless, worldwide epidemiological studies have reported that the number of individuals who debuted before 50 years old has increased [8, 9, 10] and moreover, the specificity of FOBT is quite low, being significantly higher only for advanced CRC phases (stages III–IV) [11]. In addition, if FOBT results are positive, colonoscopy is recommended, which is unnecessary in most cases where other disorders may cause rectal bleeding, involving a significant workload in hospitals. Therefore, there is an urgent need for new, more specific, and noninvasive biomarkers to enhance early detection of colorectal neoplasia.

Multiple risk factors may be involved in the development of CRC such as environmental changes, unhealthy lifestyle behaviors, physical inactivity, detrimental dietary patterns, excessive alcohol/tobacco consumption, overweight, and obesity, among others [6]. In particular, the consumption of high levels of sugar and saturated fats (typical of Western diets) has been suggested as a potential cause of the increasing incidence of CRC in young people [12]. Additionally, over the last 10 years, researchers have explored the human gut microbiota through next‐generation sequencing (NGS) technologies, demonstrating that gastrointestinal bacterial communities play an important role in the development of several pathological processes [13, 14, 15, 16, 17, 18, 19, 20], including cancer [21, 22, 23, 24, 25, 26]. Microbial taxonomic composition in the gut is directly related to and conditioned by intrinsic (e.g., age, sex, or innate and adaptive immunity) and extrinsic (e.g., local environment, diet, medication, cultural habits, physical activity or transit time) host factors [27]. Alterations in the gut microecosystem may lead to microbial imbalance (dysbiosis), promoting chronic intestinal inflammation [26, 28], tissue impairment [28, 29, 30] and the breakdown of the gastrointestinal barrier [29], which could induce the transition from colorectal polyps to carcinomas [31, 32, 33]. Therefore, intestinal microbiome disruption could be an important risk factor for CRC. Similarly, previous studies have suggested that oral microbiota dysbiosis is related to different gut diseases, including CRC [23, 30, 34, 35, 36, 37]. In fact, saliva microbiome composition seems to vary significantly between CRC patients and individuals without CRC [35]. Accordingly, over the last years, several studies have proposed gut bacteriome biomarkers for CRC diagnosed, based on the study of different patients' cohorts all over the world [35, 38, 39, 40, 41, 42, 43, 44, 45]. Microbiome‐derived biomarkers have been demonstrated to have the potential to differentiate between individuals without gut carcinoma or dysplasia from those with colorectal carcinoma or with a high risk of developing CRC, suggesting that microbiota dysbiosis occurs before, during and after the adenoma to carcinoma transition process [25, 35, 46, 47, 48, 49].

In addition, sequencing technologies and quantitative PCRs techniques identified the presence of periodontal pathogens in feces and in colon tissues of CRC patients, such as *Fusobacterium nucleatum*, *Porphyromonas gingivalis*, *Parvimonas micra*, *Peptostreptococcus stomatis* and *Actinomyces odontolyticus*, among others [21, 23, 34, 37, 43, 50]. Oral pathobionts can migrate from the oral cavity to other tissues, via circulatory system and/or directly to the colon via the gastrointestinal tract (GIT), as previously reported [21, 31, 34, 37, 51, 52] promoting inflammatory and tumorigenesis processes [52]. In the present work, we performed an in‐depth analysis of the oral and intestinal microbiome of samples obtained from a cohort of CRC patients (gingival crevicular fluid, saliva, non‐neoplastic tissues, adenocarcinoma tissues, and feces), to establish correlations between bacteria within and between niches and to provide new insights about the oral‐gut connection. Correlation analyses were performed to identify the bacterial consortia present in the oral and gut environments, including the tumor tissues. The comparison between microbiomes from CRC patients and those of healthy people allowed us to describe a specific combination of bacteria that could serve as a powerful noninvasive biomarker for CRC diagnosis.

## 2 Materials and methods

### 2.1 Patient's recruitment

**Fig. 1:**
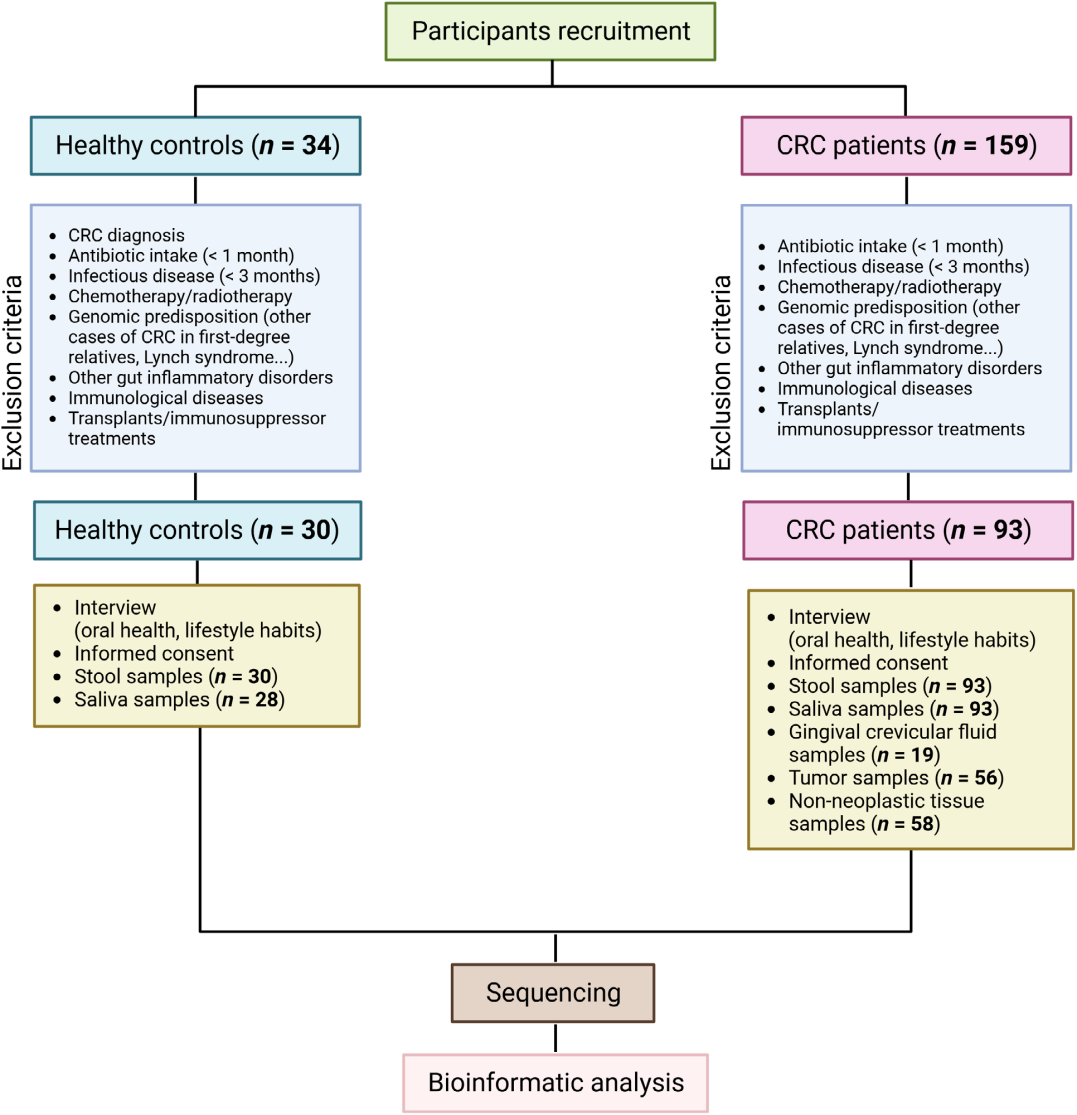
Flow chart of the study. A total of 159 patients diagnosed with colorectal cancer (CRC) at the University Hospital of A Coruña (CHUAC) were enrolled in this project. To ensure homogeneity among CRC participants, a set of exclusion criteria was established, resulting in the inclusion of only 93 out of the initial 159 patients. Concurrently, companions or couples of CRC patients, who shared similar lifestyles and ages, were invited to participate, and only 34 of them consented to join the study. Individuals constituting the healthy control cohort (non‐CRC) were subjected to the same exclusion criteria as CRC patients, with the additional requirement of not having been diagnosed with any type of cancer. Ultimately, only 30 out of the 34 individuals fulfilled these criteria. Informed consent was obtained from all participants prior to the sample collection phase. Subsequently, for microbial identification, two hypervariable regions of the 16S rRNA gene (V3‐V4) were sequenced. Bioinformatic analysis was employed to determine the bacterial diversity in each sample.

A total of 159 patients with CRC from the University Hospital of A Coruña (CHUAC) were recruited between October 2019 and May 2022. To select CRC patients for this study, several exclusion criteria were established: (a) antibiotic intake in < 1 month, (b) no infectious disease in the last 3 months, (c) chemotherapy and/or radiotherapy treatments prior to sample collection, (d) genomic predisposition to develop CRC (other cases of CRC in first‐degree relatives or Lynch syndrome among others), (e) diagnosis of other gut disorders (such as inflammatory bowel disease), (f) immunological diseases and (g) transplants and/or any immunosuppressor treatment. Based on these criteria only 93 of the initial 159 patients were included. Likewise, CRC patients' companions/couples were asked to participate in the study and only 34 agreed to participate. Individuals included in this non‐CRC control cohort followed the same exclusion criteria as CRC patients but were not diagnosed with any type of cancer. The rigorous selection process performed in our study ensured that the control group consisted of individuals who were free from cancer, making it a suitable comparison cohort for studying the microbiome in the context of CRC. Only 30 of the 34 individuals met the requirements. Informed consent was obtained from all the participants before the sample collection phase (Fig. 1).

### 2.2 Sample collection

A total of 93 samples of *ca*. 20 mL of feces (F) and 93 samples of 5 mL of unstimulated saliva (S) were collected at home by CRC patients included in the study, before starting the low‐residue diet required for laparoscopy. Thirty healthy individuals were recruited from the same samples: F (*n* = 30) and S (*n* = 28). A previous interview was conducted with all participants to recover data related to age, sex, weight, height, and lifestyle habits, such as diet or sporting activity, oral diseases, antibiotic, intake, and previous surgeries. Furthermore, the F samples were kept in the presence of 10 mL of RNAlater reagent (Thermo Fisher Scientific, Waltham, MA, USA). Gingival crevicular fluid (GCF) samples (*n* = 19) were collected only from the CRC patients group during dental check‐ups by inserting sterile paper points (ISO 30, Henry Schein, Melville, NY, USA) in the subgingival sulcus of different teeth for 30 s. Eight paper points *per* patient were obtained and placed in an Eppendorf tube containing 500 μL of RNAlater (Thermo Fisher Scientific). A total of 56 adenocarcinoma tissue samples from the colon (Ac) and 58 non‐neoplastic colon tissue samples from the surrounding areas (NM) were collected via sterile dissection during surgical resection. A total of 2 g of amoxicillin per clavulanic acid was administered to the patients at the beginning of the laparoscopic and postoperatory stages (a total of 3 doses every 8 h). All tissue samples were immediately stored in the presence of 500 μL of RNAlater reagent (Thermo Fisher Scientific) for nucleic acid extraction and sequencing. All samples were stored at −80 °C until further analysis.

### 2.3 Oral and dental check‐up

As cited above, GCF samples from CRC patients who agreed to undergo an oral examination were collected by a periodontist at the Pardiñas Medical Dental Clinic (A Coruña, Galicia, Spain). Only 19 of 93 went to the dental check‐ups. Periodontal assessment for each patient was done according to the 2017 World Workshop on the Classification of Periodontal and Peri‐Implant Diseases and Conditions [53]. The gingival condition of each patient was recorded using the Silness‐Loe gingival index [54]. The decayed, missing, and filled teeth (DMFT) index was assessed for each patient using intraoral examination and cone beam computed tomography (CBCT; Carestream Dental LLC, Atlanta, GA, USA). The presence or absence of periapical lesions was ruled out by CBCT analysis in each patient.

### 2.4 Bacterial DNA extraction

#### 2.4.1 Stool and saliva samples

Samples from both CRC and non‐CRC individuals were thawed and subsequently subjected to centrifugation (2 min, 4500 ***g***, 4 °C). Following this, each supernatant underwent a second centrifugation round (10 min, 21 000 ***g***, 4 °C). The resultant pellets were resuspended in nuclease‐free water and incubated for 1 h at 37 °C and 400 rpm in the presence of a specific enzymatic cocktail (EC). Bacterial DNA extraction from the samples was performed using the MasterPureTM Complete DNA and RNA Purification Kit (Epicentre, Madison, WI, USA). Our team has previously provided a comprehensive and detailed exposition of this methodology [55].

#### 2.4.2 Tissue and gingival crevicular fluid samples

Bacterial DNA from Ac and NM samples was extracted from 20 mg of each tissue using the AllPrep^®^ DNA/RNA Mini kit (Qiagen, Hilden, Germany). Homogenization was performed using Lysing Matrix E tubes (MP Biomedicals, St. Ana, CA, USA) and a 1600 MiniG system (Thermo Fisher Scientific). The EC was added also to this kind of samples after homogenization.

With respect to the GCF samples, a 2 min vortex was used to detach bacteria from gingival papers, which were carefully discarded afterwards. A new centrifugation (21 000 ***g***, 30 min, 4 °C) was carried out and the bacterial pellets were used to extract bacterial DNA with the AllPrep^®^ DNA/RNA Mini kit, following the manufacturer's instructions, incorporating only an additional enzymatic lysis step through the use of the EC.

For all types of samples, the final DNA was eluted and stored at −20 °C until library preparation. Negative controls for each extraction were done to avoid contaminations. All these protocols were described in greater detail in a previous work [55].

### 2.5 16S rRNA metabarcoding

Two hypervariable regions of the 16S rRNA gene (V3‐V4) were amplified by PCR using the following oligonucleotides: 5′ TCGTCGGCAGCGTCAGATGTGTATAAGAGACAGCCTACGGGNGGCWGCAG as the forward primer and 5′ GTCTCGTGGGCTCGGAGATGT GTATAAGAGACAGGACTACHVGGGTATCTAATCC as the reverse primer [55, 56].

All libraries were prepared following the Illumina 16S Metagenomic Sequencing Library Preparation protocol (Illumina, San Diego, CA, USA). All sequencing processes were conducted using paired‐end Illumina MiSeq v3 reagent kits (2 × 300) (Illumina). Library sizes were assessed with a 2100 Bioanalyzer (Agilent Technologies, St. Clara, CA, USA). This sequencing protocol was in‐depth detailed in a previous publication [55].

### 2.6 Bioinformatic analysis

The quality of all FASTQ files generated from 16S rRNA gene sequencing was checked using fastqc [57]. Afterwards, sequences were analyzed using qiime2 (version 2021.11) [58] on a per sequencing run basis, utilizing DADA2 to trim, denoise, correct sequencing errors and remove chimeras, resulting in several tables of Amplicon Sequence Variants (ASVs) [59]. The resulting features from each sequencing run were then merged and collapsed into a single feature table and classified using the SILVA 138 99% reference database [60] through QIIME2. Afterwards, r (version 4.1) [61] and phyloseq (version 1.36.0) [62] were used to create a Phyloseq object from QIIME 2 results, to process it and to clean it. Mainly, filtering ASVs to those from the bacterial kingdom, subtracting the raw count of ASVs that appeared in control samples from the rest on a per sequencing run basis and removing ASVs in specific genera that are typically involved in reagent contamination [63]. Additionally, taxonomy levels were propagated (e.g., An ASV that was classified at the genus level but not classified at the species level had their last known level propagated, filling the species level with Genus_NA). To calculate the mean relative abundance for each type of sample the cleaned and rarified Phyloseq was used. Filters composed of a minimum abundance of 0.01% and minimum prevalence of 30% were applied, firstly in a per biosample basis (each biosample can be sequenced multiple times) and then in a per group basis (e.g., feces of CRC patients), obtaining the mean relative abundance in multiple taxonomy levels. Alpha and beta diversity analyses were also performed through R and Phyloseq. Differential abundance analysis (DAA) was performed using r package ancom‐bc (version 2.0.1) [64] on the cleaned, non‐rarified Phyloseq object, adjusting the *P*‐values by the Holm‐Bonferroni method [65] and using a prevalence cut of 30%, except in the test of agglomerations of oral genera, in which prevalence filter was removed to show every genus of interest.

To perform the co‐occurrence study raw counts were normalized with ANCOM‐BC [64] and species with <0.01% of mean abundance or in <30% of the samples were filtered out. Paired samples (S, GCF, NM, and Ac from the same CRC individuals) were used to study the intra‐ and inter‐niche correlations at the bacterial level. To assess the correlations among the bacteria in the oral samples, unsupervised sPCA from mixomics r package was performed. To elucidate the associations of oral bacteria in tumors a multivariate analysis (sPLS‐canonical) from the mixomics r package was applied using the normalized dataset of all bacterial counts from the tissue sample and a subset of the oral‐associated bacteria present in the samples [66].

Normalized data from 47 MN tissues were used as input for Dirichlet Multinomial Mixtures (DMM) algorithm [67] to identify the optimal number of clusters (tissue enterotypes) based on Laplace approximation. Subsequently, Bray Curtis distances were calculated and for graphical visualization, an ordination technique was performed with non‐metric multidimensional scaling (NMDS) method [68]. Taxonomic differences among enterotypes were assessed using Wilcoxon rank sum paired tests and canonical correlation analysis (CCA) was used to plot these differences using r [61].

Logarithmic regression models were used to evaluate the discriminatory capacity of bacteria as biomarkers in feces. Accuracy was evaluated using receiver operating characteristic (ROC) curves and the area under the curve (AUC) was calculated and validated using the leave‐one‐out cross‐validation (LOOCV) method. Additionally, the bootstrapping algorithm implemented in the Boruta R library [69] was used to blind the selection of additional biomarkers. The analysis was performed at the genus level.

For more information on the bioinformatics methods used in this work please see the scripts referenced in the data availability statement of the manuscript.

### 2.7 External validation of the proposed biomarkers

We executed a methodically process to preliminarily validate our proposed bacterial biomarkers. This involved an examination of three distinct 16S rRNA datasets (Table 1) obtained from previous studies [45, 70, 71] using the bioinformatic tools described in the present study (see Data availability statement). For each dataset, we analyzed fecal samples from CRC and healthy individuals using the r package ancom‐bc and setting the prevalence threshold at 10%.

**Table 1:** External 16S rRNA datasets used in the present study for the validation of our proposed bacterial biomarkers.

Dataset authors	Cohort location	Bioproject/link
Baxter *et al*. 2016	USA	PRJNA290926
Zeller *et al*. 2014	France	ERP005534
Zackular *et al*. 2014	Canada, USA	http://www.mothur.org/MicrobiomeBiomarkerCRC

### 2.8 Ethics approval

This study was adhered to the standards of clinical practice and research regulations (Law of Biomedical Research 14/2007), in agreement with the Declaration of Helsinki and the Convention on Human Rights and Biomedicine. Compliance with the protection of non‐public personal data of all those involved within the RGPD – UE 2016/679, LOPDGDD 3/2018 Law 41/2002 and its implementing regulations, Royal Decree 1720/2007, were enforced. This project (PI20/00413), granted by Carlos III Health Institute (ISCIII; Spain), was supervised by the local ethics committee, the Research Ethical Committee of Galicia (code CEIm‐G 2018/609, Galicia, Spain), and by the Spanish Agency for Medicines and Healthcare Products (AEMPS) for the use of CRC patients' samples from CHUAC (A Coruña, Galicia, Spain). Informed consent for Biobank (CHUAC, A Coruña, Galicia, Spain, UNE‐EN ISO 9001‐2015) was signed by all individuals grouped in this study. Anonymized clinical data used during the study for CRC patients was obtained from the Galician Health Service (SERGAS). All individuals recruited in this project (CRC and non‐CRC) signed a formal consent form for the publication of scientific and clinical results in scientific articles.

## 3 Results

### 3.1 CRC patients and healthy controls characteristics

**Fig. 2:**
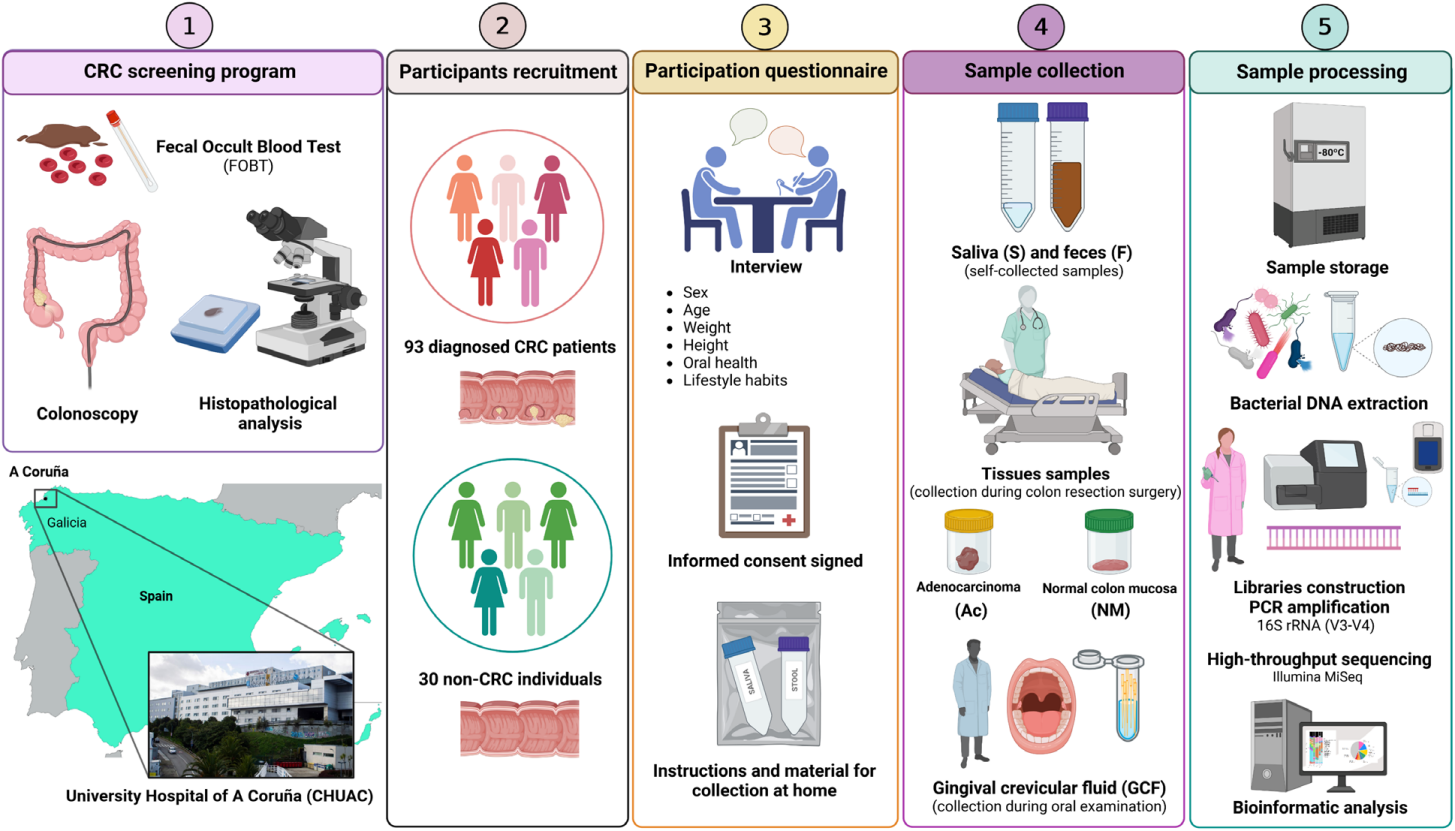
Schematic representation of the workflow during the present study. All colorectal cancer (CRC) patients involved in this study were diagnosed and treated at the University Hospital of A Coruña (CHUAC; Galicia, Spain). CRC diagnosis was based on: (1) positive colonoscopy for colorectal neoplasia confirmed by histopathological analysis of biopsied tissues or (2) through CRC screening programs consisting of a positive fecal occult blood test (FOBT) followed by a positive colonoscopy validated by histopathological analysis. The cohort for the study consisted of 93 patients diagnosed with CRC and 30 healthy individuals without any digestive disorders (non‐CRC). All the participants completed a questionnaire and collected saliva (S) and fecal (F) samples at home. Tissue samples such as normal colorectal mucosa tissue (NM) and adenocarcinoma tissue (Ac) of CRC patients were collected by the surgery team after colon laparoscopic resection at CHUAC. Besides, gingival crevicular fluid samples (GCF) were collected at Pardiñas Medical Dental Clinic (A Coruña, Galicia, Spain) during an oral examination. Finally, all different‐nature samples were sent to the microbiology laboratory at CHUAC where they were processed, sequenced, and analyzed using different bioinformatic tools.

A total of 93 patients with CRC and 30 healthy individuals (non‐CRC) were included in the present study. CRC diagnosis was confirmed by colonoscopy and histopathological analysis in all cases. The characteristics of both groups are summarized in Tables 1 and 2. Figure 2 summarizes the workflow of this study.

**Table 2:** Characteristics of the colorectal cancer patients' cohort (CRC, *n* = 93) and the control group (non‐CRC; *n* = 30).

	Colorectal cancer (CRC)	Control (non‐CRC)	Total (CRC + non‐CRC)	*P‐*value
Sex, *n* (%)				
Females	36 (38.71)	23 (76.67)	59 (47.97)	0.020
Males	57 (61.29)	7 (23.33)	64 (52.03)	
Age (years), median				
Females	65	63	65	0.107
Males	70	64	67	0.053
Height (cm), median				
Females	170	160	160	0.984
Males	169	167.5	169	0.281
Weight (kg), median				
Females	71	67	68	0.491
Males	77	82	77.5	0.635
Location, *n* (%)				
Cecum	6 (6.45)	–	6 (6.45)	
Ascending colon	29 (31.18)	–	29 (31.18)	
Hepatic flexure	1 (1.07)	–	1 (1.07)	
Transverse colon	3 (3.22)	–	3 (3.22)	
Splenic flexure	2 (2.15)	–	2 (2.15)	–
Descending colon	22 (23.65)	–	22 (23.65)	
Sigmoid colon	18 (19.35)	–	18 (19.35)	
Rectum	10 (10.75)	–	10 (10.75)	
Undetermined	2 (2.15)	–	2 (2.15)	
CRC stage (TNM), %				
T [1, 2, 3, 4]	T1 (17.20); T2 (12.90); T3 (62.36); T4 (5.38)	–	T1 (17.20); T2 (12.90); T3 (62.36); T4 (5.38)	
N [0–5]	N0 (64.51); N1 (17.20); N2 (7.53); N3 (2.15); N4 (2.15); N5 (2.15)	–	N0 (64.51); N1 (17.20); N2 (7.53); N3 (2.15); N4 (2.15); N5 (2.15)	–
M [0–3]	M0 (92.47); M1 (2.15); M2 (2.15); M3 (1.07)	–	M0 (92.47); M1 (2.15); M2 (2.15); M3 (1.07)	
Undetermined	2.06	–	2.06	
Bristol Stool Scale, *n* (%)				
Type 1	0 (0)	0 (0)	0 (0)	0.399
Type 2	8 (8.60)	3 (10)	11 (8.94)	
Type 3	10 (10.76)	1 (3.33)	11 (8.94)	
Type 4	38 (40.86)	19 (63.34)	57 (46.35)	
Type 5	8 (8.60)	3 (10)	11 (8.94)	
Type 6	10 (10.75)	1 (3.33)	11 (8.94)	
Type 7	5 (5.38)	1 (3.33)	6 (4.88)	
Undetermined	14 (15.05)	2 (6.67)	16 (13.01)	
Periodontal disease (PD), *n* (%)				
No	33 (35.48)	14 (46.67)	47 (38.21)	0.535
Yes	54 (58.07)	15 (50)	69 (56.10)	
Undetermined	6 (6.45)	1 (3.33)	7 (5.69)	
Physical activity, *n* (%)				
No	44 (47.31)	21 (70)	65 (52.85)	0.083
Yes	40 (43.01)	7 (23.33)	47 (38.21)	
Undetermined	9 (9.68)	2 (6.67)	11 (8.94)	
Alcohol consumption, *n* (%)				
No	54 (58.06)	21 (70)	75 (60.98)	0.560
Yes	30 (32.26)	7 (23.33)	37 (30.08)	
Undetermined	9 (9.68)	2 (6.67)	11 (8.94)	
Tobacco consumption, *n* (%)				
No	70 (75.27)	22 (73.33)	92 (74.8)	0.788
Yes	14 (15.05)	6 (20)	20 (16.26)	
Undetermined	9 (9.68)	2 (6.67)	11 (8.94)	
Caffeine consumption, *n* (%)				
No	66 (70.97)	19 (63.93)	85 (69.11)	0.511
Yes	18 (19.35)	9 (29.40)	27 (21.95)	
Undetermined	9 (9.68)	2 (6.67)	11 (8.94)	
Sleep disorders, *n* (%)				
No	58 (62.37)	23 (76.67)	81 (65.85)	0.280
Yes	24 (25.81)	6 (20)	30 (24.39)	
Undetermined	11 (11.82)	1 (3.33)	12 (9.76)	
Environment (most habitual), *n* (%)				
City	31 (33.33)	18 (60)	49 (39.84)	0.551
Countryside	45 (48.39)	8 (26.67)	53 (43.09)	
Both	8 (8.60)	3 (10)	11 (8.94)	
Undetermined	9 (9.68)	1 (3.33)	10 (8.13)	

A total of 377 samples were sequenced and analyzed using different bioinformatic procedures. In the CRC group, 93 fecal samples (F), 93 saliva samples (S), 19 gingival crevicular fluid samples (GCF), 58 normal colorectal mucosa samples (NM) and 56 adenocarcinoma samples (Ac) were obtained. For the non‐CRC group, 30 F and 28 S samples were processed and analyzed. The colon distribution of the different Ac samples studied in the CRC patients revealed that 31.18% were located in the ascending colon, 23.65% in the descending colon and 19.35% in the sigmoid colon (Table 2). Most of the CRC patients of this study were diagnosed in advanced stages, being the 62.36% of them in the T3 stage and 5.38% in the T4 stage (Table 2). Nevertheless, 64.51% of the CCR patients had no lymph node invasion and 92.47% of the patients had no distant metastasis (Table 2). Interestingly, a high percentage of patients with CRC (58.07%) were admitted having oral disorders to the questionnaire (e.g., halitosis, gingivitis, periodontitis, and tartar on teeth or caries, among others). In contrast, only 23.33% of the individuals in the non‐CRC control group presented dental or gum disorders (Table 2). Specifically, among CRC patients who agreed to attend the oral check‐up, 89.47% had periodontal disease in early or advanced stages (Table 3). Further characteristics of the studied cohorts (CRC and non‐CRC) are summarized in Tables 2 and 3.

**Table 3:** Oral health characteristics of the colorectal cancer (CRC) patients who attended the oral and dental check‐up.

Colorectal cancer (CRC)				
			*n*	%
Periodontal disease (PD)	Gingivitis	IA	1	5.26
		IB	1	5.26
	Initial	IIA	3	15.79
	Periodontitis	IIB	3	15.79
	Mild	IIIA	4	21.06
	Periodontitis	IIIB	1	5.26
	Progressive	IVA	0	0
	Periodontitis	IVB	3	15.79
	–	No PD	2	10.53
		No teeth^a^	1	5.26
Gingival Index^b^		0.1–1	7	36.84
		1.1–2	10	52.64
		2.1–3	1	5.26
		No teeth^a^	1	5.26
Tooth loss (number of missing teeth)		1–5	9	47.36
		6–10	4	21.06
		11–15	4	21.06
		> 15	1	5.26
		No teeth^a^	1	5.26
Caries Index (DMFT Index: decayed, missing, filled)		1–10	4	21.06
		11–20	12	63.15
		> 20	2	10.53
		No teeth^a^	1	5.26
Periapical lesion		No	14	73.68
		Yes	5	26.32

a No teeth: colorectal cancer (CRC) patient with completely missing teeth due to acute periodontitis (dental implants only).

b Gingival Index: Level 0.1–1: Mild degree of gingival inflammation, slight gingival color change, no bleeding. Level 1.1–2: Moderate degree of inflammation, gingival reddening and swelling, gingival bleeding on probing and pressure. Level 2.1–3: Strong level of inflammation, intense gingival reddening and swelling, plentiful bleeding, and the possibility of ulceration.

### 3.2 Microbiome composition of CRC patients

Microbiome analysis of different samples obtained from a cohort of patients with CRC was performed (Fig. 2). To achieve this, the S and F samples from CRC patients were compared with samples from non‐CRC individuals. The median number of reads per sample across sequencing runs was 49 737 (21 million reads in total), decreasing to a median of 32 104 (13 million reads in total) after quality control and DADA2 processing. This resulted in 15 213 ASVs with a median length of 418 bp.

**Fig. 3:**
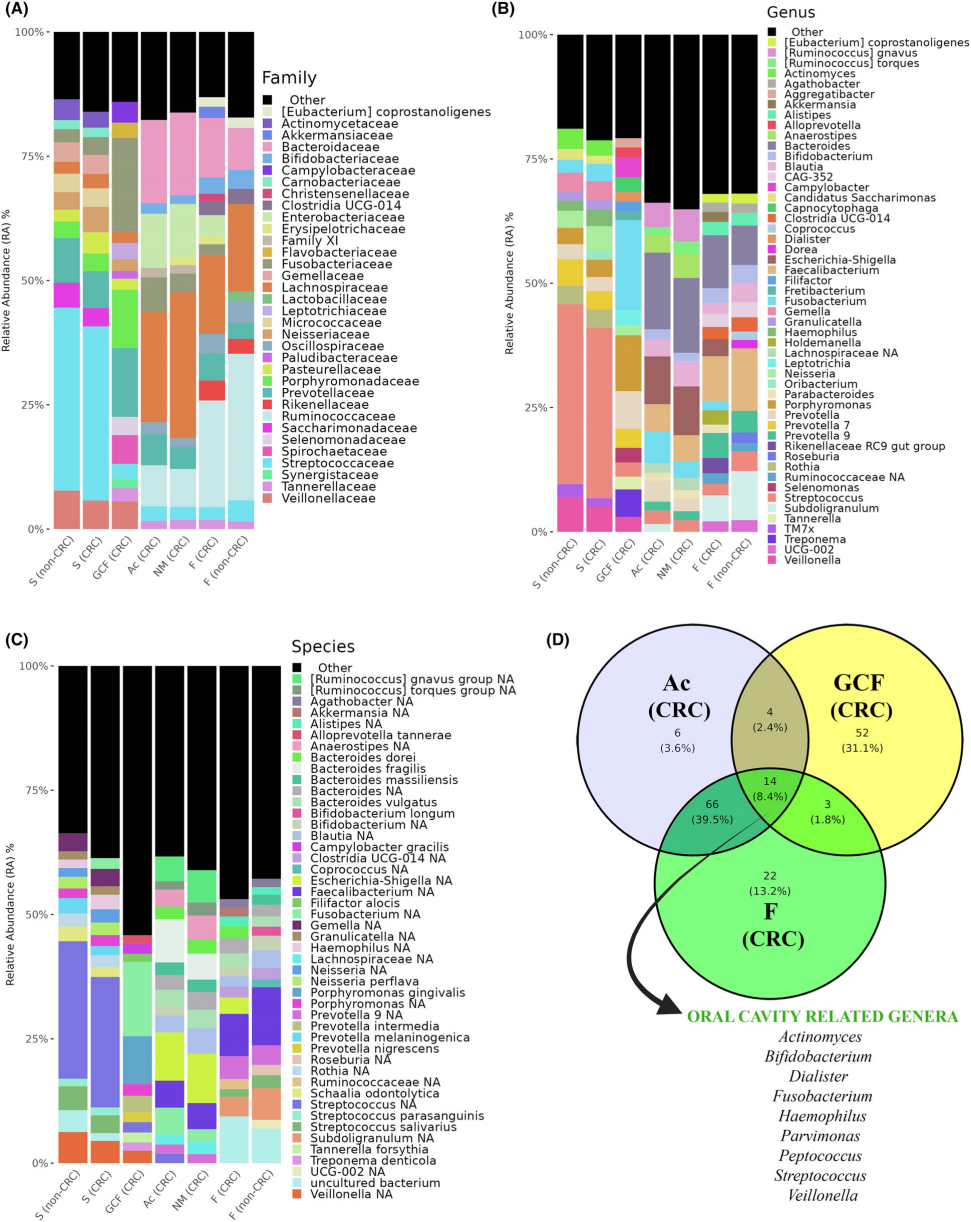
Bacteriome landscape of the different‐nature samples obtained from colorectal cancer patients (CRC) and healthy individuals without any digestive disorders (non‐CRC) analyzed by 16S rRNA Illumina sequencing. Barplots show the relative abundance (RA) at the family level (A), genera level (B) and species level (C), with a prevalence filter of 30%. The panel D shows a Venn diagram indicating the number of bacterial genera detected in each of the samples, as well as the number of genera common to all the samples with a list of oral bacteria. Samples analyzed: saliva (S) and feces (F) from both non‐CRC and CRC individuals; gingival crevicular fluid (GCF), adenocarcinomas (Ac), and normal colorectal mucosa tissues (NM) from CRC patients.

No significant differences in bacterial profiles were detected between saliva samples from CRC patients and healthy individuals, when 16S rRNA gene sequencing was done. Nevertheless, regarding the oral cavity microbiome of CRC patients, important periodontal pathobionts were identified specifically in GCF samples (Fig. 3), being the most represented: *Fusobacterium* sp. (15%), *Porphyromonas gingivalis* (9.48%), *Prevotella intermedia* (3.20%), *Prevotella nigrescens* (2.10%), *Tannerella forsythia* (2.03%), *Alloprevotella tannerae* (1.72%), *Treponema denticola* (1.65%) and the emerging periodontal pathogen *Filifactor alocis* (1.58%) (Fig. 3C).

**Fig. 4:**
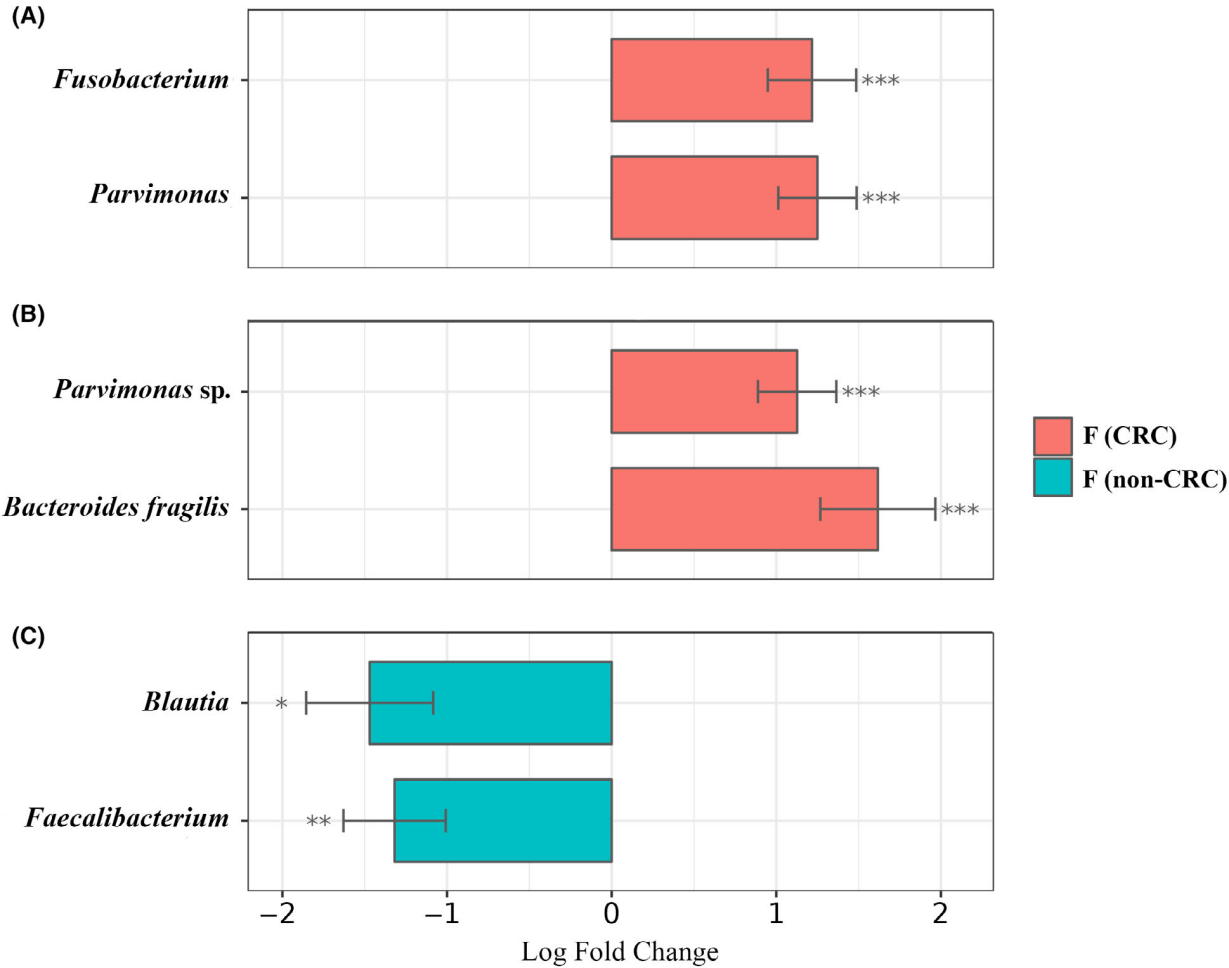
Differential abundance analysis (DAA) of the bacteriomes of fecal samples (F) from colorectal cancer (CRC) patients and healthy individuals without any digestive disorders (non‐CRC). Analyses were made at the genus (A), species (B) and amplicon sequencing variants (ASVs) (C) levels. Effect size (log fold change), standard error and adjusted *P‐*values for each entry were obtained using the ANCOM‐BC method, with a prevalence filter of 30% and subsequent Holm‐Bonferroni statistical test. Only effect sizes with adjusted *P‐*values < 0.05 are shown (***: < 0.001, **: < 0.01, *: < 0.05).

Regarding the gut microbiome, and focusing on F samples, *Ruminococcaceae*, *Lachnospiraceae*, and *Bacteroidaceae* were the most abundant families in the two groups (CRC and non‐CRC) as shown in Fig. 3A. However, *Prevotellaceae*, *Enterobacteriaceae* and *Rikenellaceae* were more abundant in the CRC group than in the non‐CRC group (Fig. 3A). Interestingly, the *Fusobacteriaceae* family was significantly over‐enriched in the CRC samples (adjusted *P‐*value < 0.001). In contrast, in the F samples from the healthy group, the families *Oscillospiraceae*, *Streptococcaceae* and *Bifidobacteriaceae* were more represented compared to F samples in CRC patients (Fig. 3A). Consequently, *Fusobacterium* and *Parvimonas* genera in fecal samples of CRC patients were significantly over‐abundant (adjusted *P*‐values < 0.001 in both cases) compared to F samples of the non‐CRC group (Fig. 4). Notably, when taking sex into account these differences were still visible, being more abundant in both males and females diagnosed with CRC. More specifically, at the species level, *Parvimonas* sp., an important periodontal pathogen, and *Bacteroides fragilis* were significantly enriched (adjusted *P*‐values < 0.001 in both cases) in F samples from CRC patients when compared to F samples from non‐CRC individuals (Fig. 4). In contrast, focusing on ASVs level analysis, *Blautia* sp. and *Faecalibacterium* sp. were significantly more abundant in F samples from the non‐CRC control group (adjusted *P*‐values of < 0.05 and < 0.01, respectively) (Fig. 4).

**Fig. 5:**
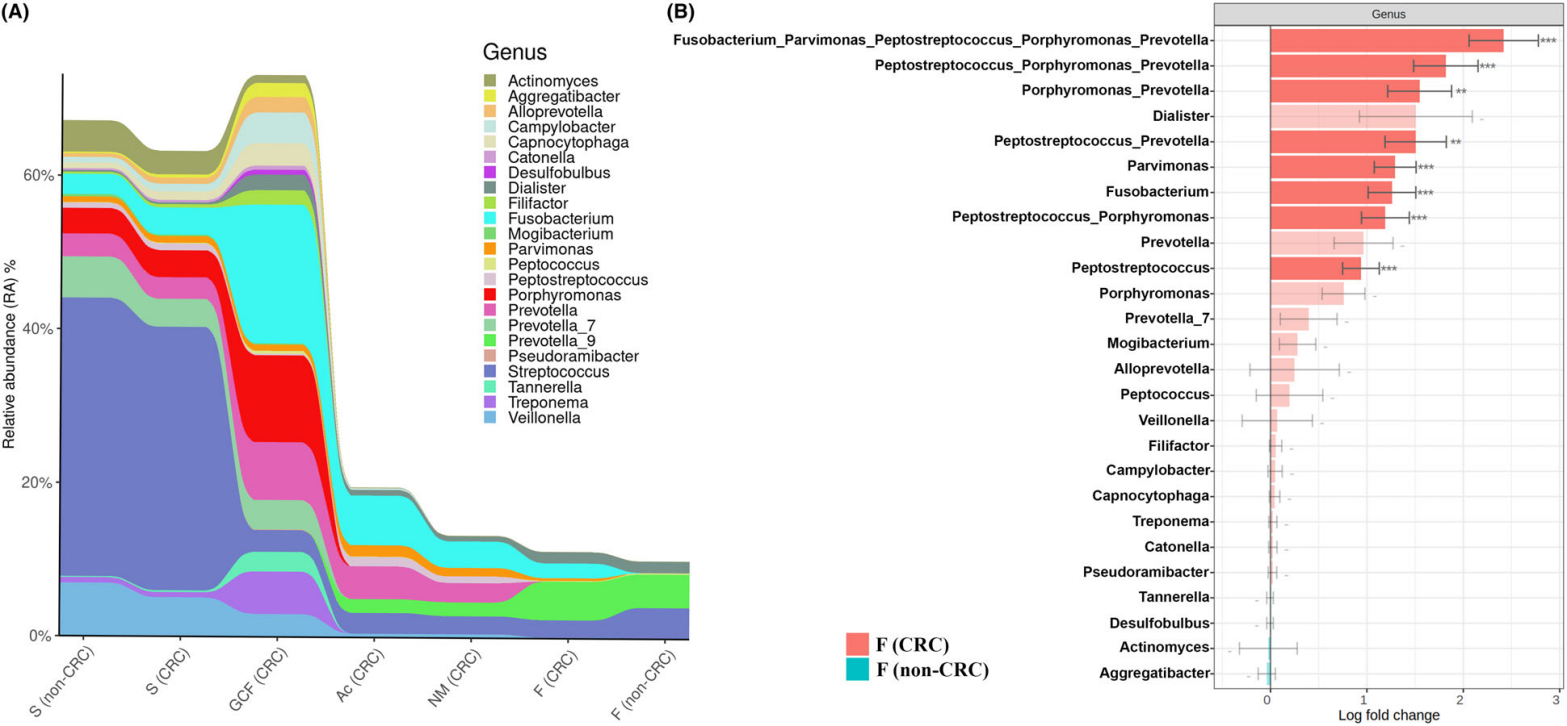
Landscape of oral‐related bacteria among different‐nature samples of colorectal cancer patients (CRC) and healthy individuals without any digestive disorders (non‐CRC). (A) Alluvial barplot of oral‐related bacterial and their relative abundance (RA) among the different samples: saliva (S) from non‐CRC and CRC individuals, gingival crevicular fluid (GCF) from CRC patients, adenocarcinomas (Ac) from CRC patients, normal colorectal mucosa tissues (NM) from CRC patients and feces (F) from both non‐CRC and CRC participants. (B) Differential abundance analysis (DAA) of groups of amplicon sequencing variants (ASVs) from typical oral‐related genera obtained from feces (F) from CRC and non‐CRC individuals. Effect size (log fold change), standard error and adjusted *P‐*values for each entry were obtained using the ANCOM‐BC method and subsequent Holm‐Bonferroni statistical test (*P‐*values ***: < 0.001, **: < 0.01, −: > 0.1). No prevalence cut was used for this analysis in order to show unsignificant entries belonging to oral‐related bacteria.

When the analysis was focused only on oral‐related microorganisms, the genera *Fusobacterium* and *Parvimonas* appeared across all types of samples except in F of non‐CRC individuals (Fig. 5A), showing more abundance in GCF, Ac, NM and F of CRC patients (Fig. 5A). Differential abundance analysis of specific combinations of ASVs integrating typical oral‐related genera was conducted using data obtained from F samples from CRC and non‐CRC individuals. The data revealed that the group formed by *Parvimonas*, *Fusobacterium*, *Prevotella*, *Peptostreptococcus* and *Porphyromonas* was over‐abundant in CRC compared to non‐CRC fecal samples (Fig. 5B). However, only *Fusobacterium*, *Parvimonas* and *Peptostreptococcus* showed significant differences when used alone (Fig. 4).

In addition, the microbiome differences between the Ac and NM samples obtained from the same individuals with CRC were analyzed (Fig. 3). At the genus level, *Fusobacterium* and *Prevotella* were over‐represented in Ac samples compared to NM samples from the same individual. In contrast, the *Ruminococcus gnavus* and *torques* groups, *Anaerostipes* and *Blautia* were more abundant in NM than in Ac (Fig. 3B). At the species level, the results demonstrated that *Bacteroides fragilis* was enriched in Ac tissue (8.7%) when compared to NM samples (5%) of CRC patients, in accordance with results observed in F samples of CRC patients when compared to samples from non‐CRC individuals (Fig. 3C).

No significant differences in alpha diversity among the CRC and non‐CRC groups for F and S samples were detected. When quantifying the influence of covariables in beta‐diversity measures, F appeared to be influenced by patient group (CRC, non‐CRC; *P*‐value < 0.01) and age (*P*‐value < 0.05), but not sex. Analysis of Ac samples revealed significant influences of age (*P*‐value < 0.05) and sex (*P*‐value < 0.05), but not of the location (right *vs*. left colon), tumor size, metastasis, or lymph node affectation. Additionally, S samples were not influenced by patient group, age, or sex.

### 3.3 Bacterial co‐occurrence

**Fig. 6:**
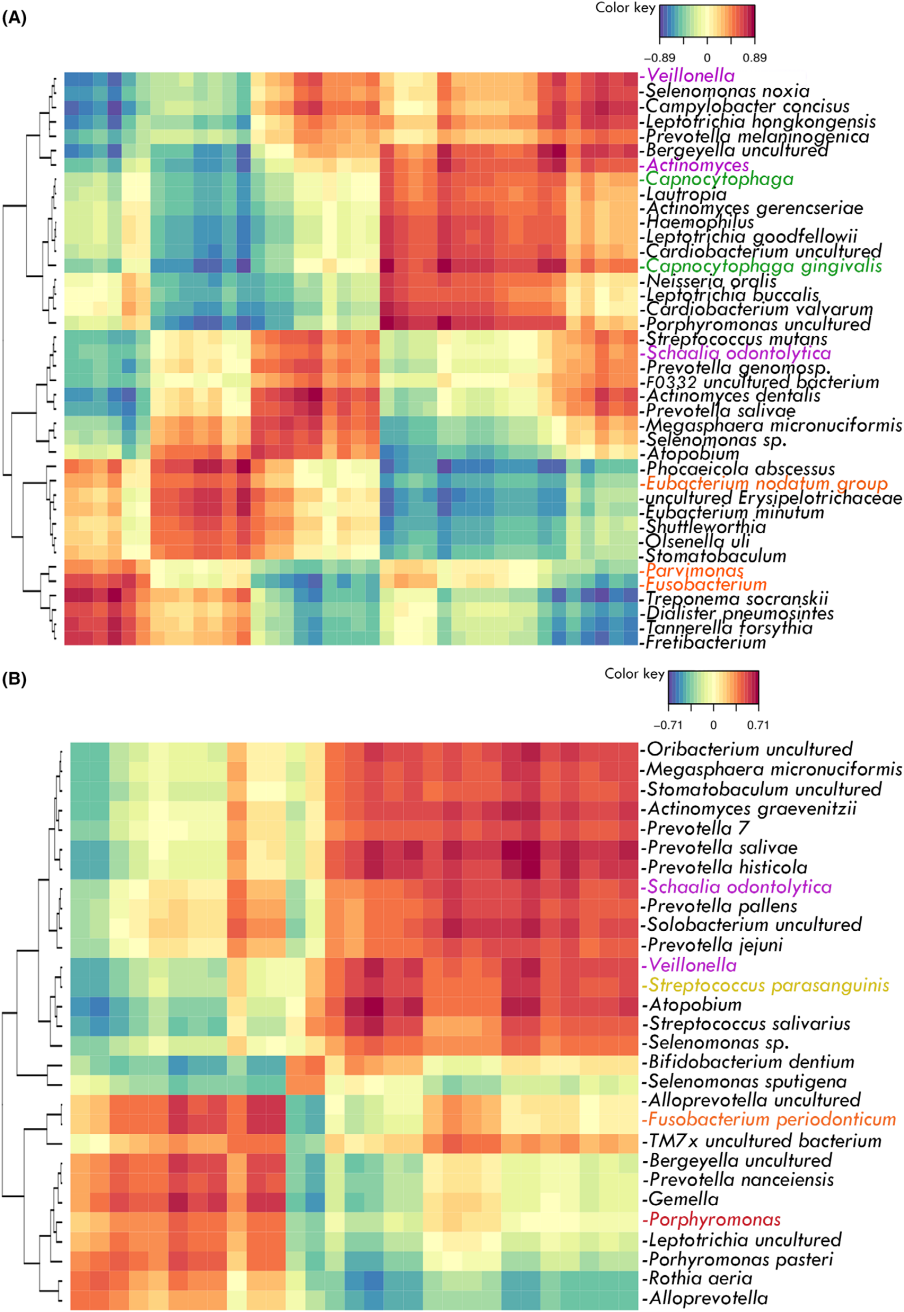
Heat map showing the associations found among bacteria in the oral cavity of colorectal cancer (CRC) patients. (A) Gingival crevicular fluid samples (GCF) of CRC patients. (B) Saliva samples (S) of CRC patients. Species belonging to the Socransky complexes (red, orange, green, yellow, blue, and purple) are marked with the corresponding color.

To corroborate the hypothesis that oral microbes translocate in complex multispecies clusters, a correlation study was conducted. Paired S, GCF, NM, and Ac samples from the same CRC individuals were used to study intra‐ and interniche bacterial correlations. In GCF samples from CRC patients, members of the red and orange Socransky complexes, well‐known as late colonizers and periodontopathogens, including *Fusobacterium*, *Parvimonas*, and *Tannerella forsythia*, were positively correlated with each other. Members of the green and purple complexes, known as early colonizers and health‐associated, also clustered together (Fig. 6A). Interestingly, in S samples of CRC patients, *Fusobacterium periodonticum* correlated positively with members or the red complex (i.e. *Porphyromonas*) as well as with other facultative anaerobes (i.e., *Gemella*) and to a lesser extent with aerobes (i.e., *Rothia aeria*) (Fig. 6B).

**Fig. 7:**
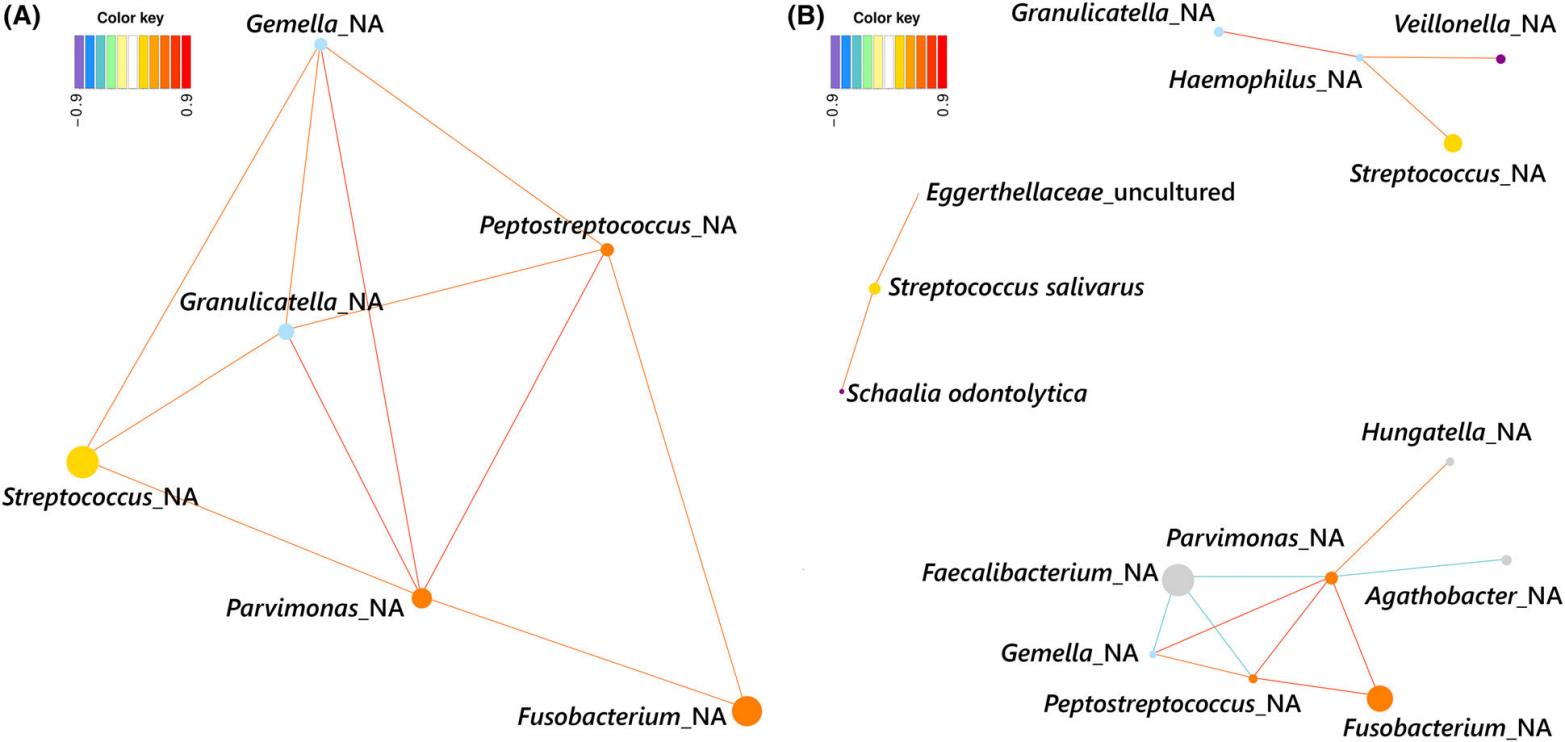
Networks among oral bacteria present in colon tissue of colorectal cancer (CRC) patients. (A) Non‐neoplastic colon tissue (NM). (B) Colorectal adenocarcinoma tissue (Ac). Edge color corresponds to correlation strength, shown in the color key. Color of nodes corresponds to Socransky complexes color and, additionally, light blue was used for other oral‐related species and gray for gut associated species. Size of nodes was related to mean abundance in tissue.

**Fig. 8:**
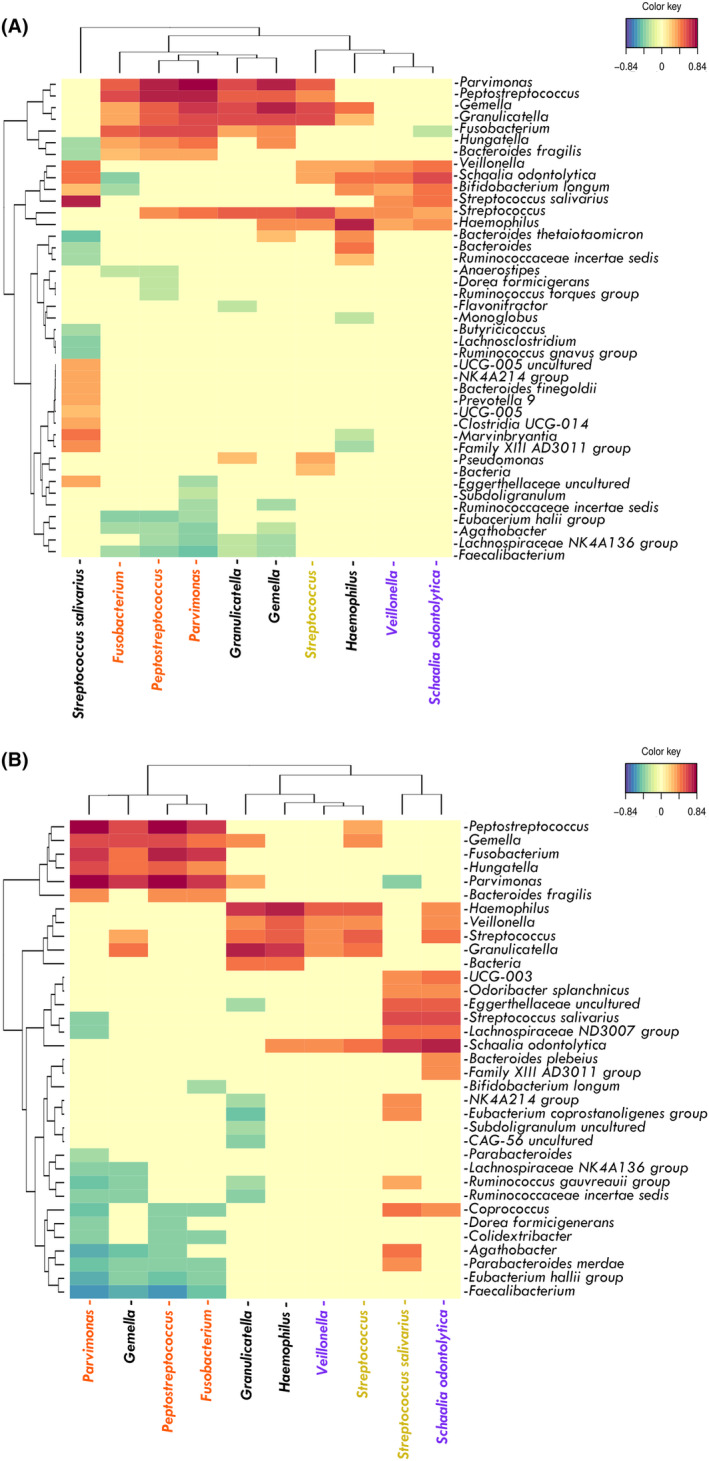
Heat map of associations between oral‐related bacteria and all the bacteria present in intestinal samples of colorectal cancer (CRC) patients. (A) Non‐neoplastic colon tissue (NM). (B) Colorectal adenocarcinoma tissue (Ac). Species belonging to the Socransky complexes were marked with the corresponding color.

When studying the correlations of oral species in NM samples from CRC patients, common patterns with S samples were detected. In this niche, proteolytic species from the orange complex (*Fusobacterium*, *Parvimonas*, and *Peptostreptococcus*) and facultative anaerobes (*Gemella*, *Granulicatella*, and *Streptococcus*) were positively correlated (Figs 7A and 8A). In the Ac samples, the same proteolytic species (*Fusobacterium*, *Parvimonas* and *Peptostreptococcus*) clustered together and had little or no correlation with facultative anaerobes (apart from *Gemella*), resembling the subgingival niche. It is worth mentioning that in tumors, the proteolytic oral cluster also correlated with intestinal microorganisms, such as unclassified *Hungatella* and *Bacteroides fragilis* (positively) or *Agathobacter* and *Faecalibacterium* (negatively) (Figs 7B and 8B). Therefore, oral proteolytic bacteria have a correlation pattern in the gut, which seems to be a pattern found in oral niches.

**Fig. 9:**
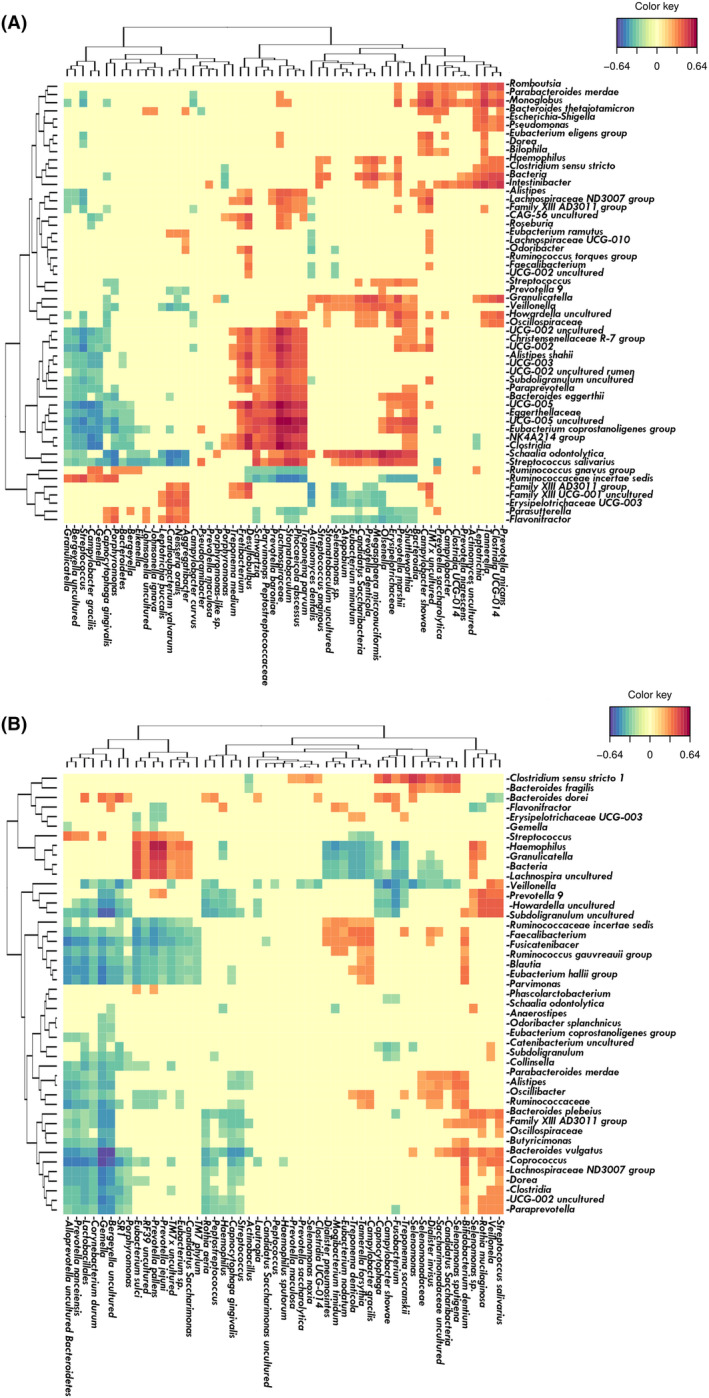
Heat map of associations between bacteria in the oral cavity and the tumor of colorectal cancer (CRC) patients. (A) Gingival crevicular fluid (GCF) and adenocarcinoma (Ac) paired samples. (B) Saliva (S) and Ac paired samples.

Finally, the correlation between bacteria in saliva or subgingival fluids and their presence in tumor tissue was analyzed, and no strong correlations were found. In fact, only *Gemella* and *Veillonella* correlated positively with itself when comparing the Ac and S samples (Fig. 9). Therefore, these results suggest that the levels of oral bacteria in the gut are not related to their corresponding levels in the oral cavity.

### 3.4 Gut enterotypes and colonization of oral bacteria

**Fig. 10:**
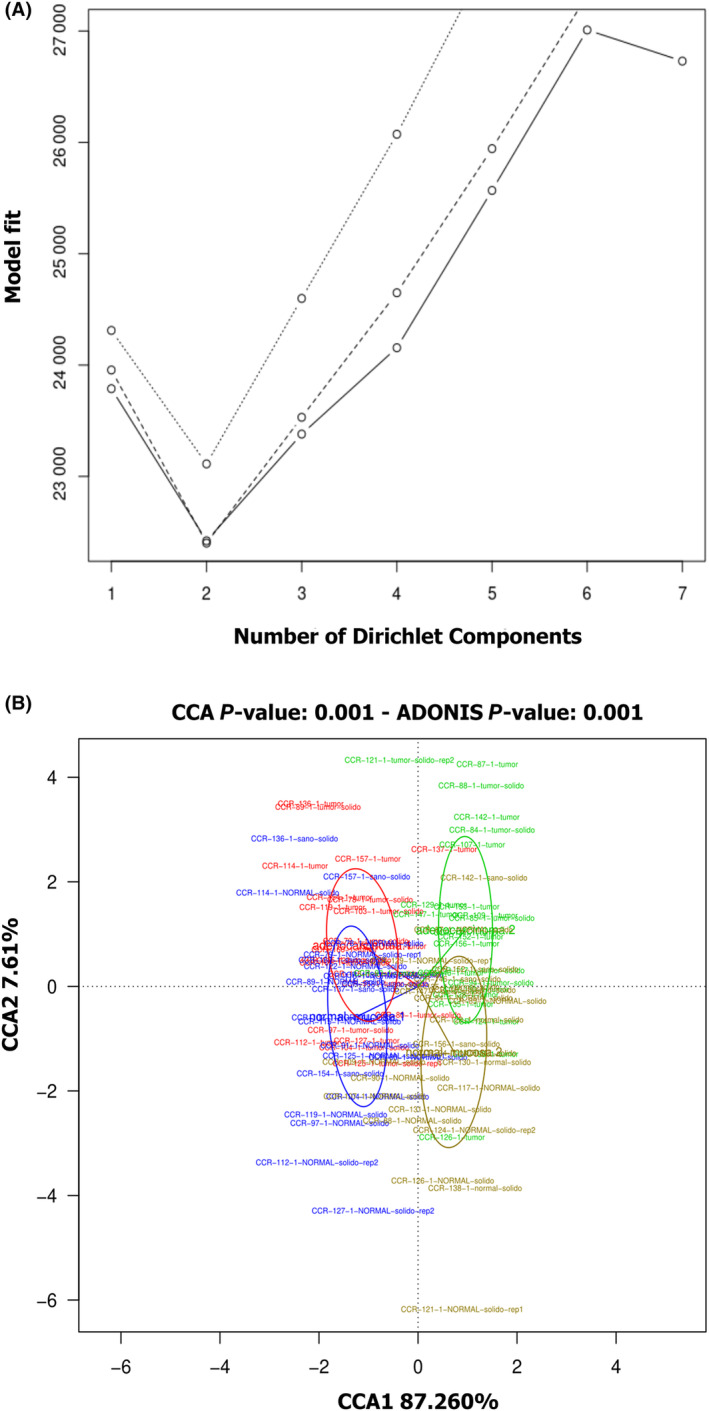
Tissue enterotypes analysis in colorectal cancer (CRC) patients. (A) Dirichlet Multinomial Mixtures (DMM) was used to infer the optimal number of community types in non‐neoplastic colon tissue samples (NM). Model fit was measured by Akaike's Information Criterion (aic) (dotted line), Bayesian Information Criterion (bic) (dashed line) and Laplace approximation (lplc) (solid line). (B) NM samples from both enterotypes (enterotype 1: blue, enterotype 2: yellow) and their corresponding adenocarcinoma sample (Ac) (enterotype 1: red, enterotype 2: green) were represented with a canonical correlation analysis (CCA) plot.

To evaluate whether certain bacterial communities in the colon are more likely to interact or facilitate colonization by oral bacteria, we analyzed the distinct enterotypes in NM samples. The DMM algorithm identified the optimal number of enterotypes in the NM tissues as two based on the Laplace approximation and NMDS ordination (Fig. 10A). Although some overlapping was observed between the clusters, the differences among them were significant. Indeed, the CCA analysis performed for the two possible enterotypes in the control/affected tissue pairs showed a higher distance among the enterotypes than among the control/affected tissue pairs (Fig. 10B). Patients classified in enterotype 1 or 2 did not differ in the clinical parameters evaluated (age, sex, tumor status, or location) (Table 4).

**Table 4:** Clinical parameters of patient classified by tissue cluster (enterotype).

	Cluster‐1	Cluster‐2	*P*‐value
Localization^a^			
Right	14	11	0.15
Left	9	13	
Tumor size			
T1	2	4	0.19
T2	4	2	
T3	16	17	
T4	1	0	
Spread to lymph nodes			
N0	16	12	0.14
N > 0	7	12	
Metastasis			
M0	21	23	0.45
M > 0	2	1	
Sex			
Male	17	16	0.5
Female	6	8	
Age			
Mean	68.78	66.29	0.15

a The right colon refers to: the cecum, ascending colon, hepatic flexure and transverse colon. The left colon refers to: the splenic flexure, descending colon, sigmoid colon and rectum.

Differences in the bacterial composition were evaluated according to the enterotypes assigned to the tumor tissues. A total of 39 species were found at significantly different levels between tumors of enterotype 1 and 2. Among them, only an uncultured *Fusobacterium* could potentially have an oral origin and is not one of the most prevalent or abundant.

### 3.5 Determination of the best bacterial consortium for CRC diagnostics

**Fig. 11:**
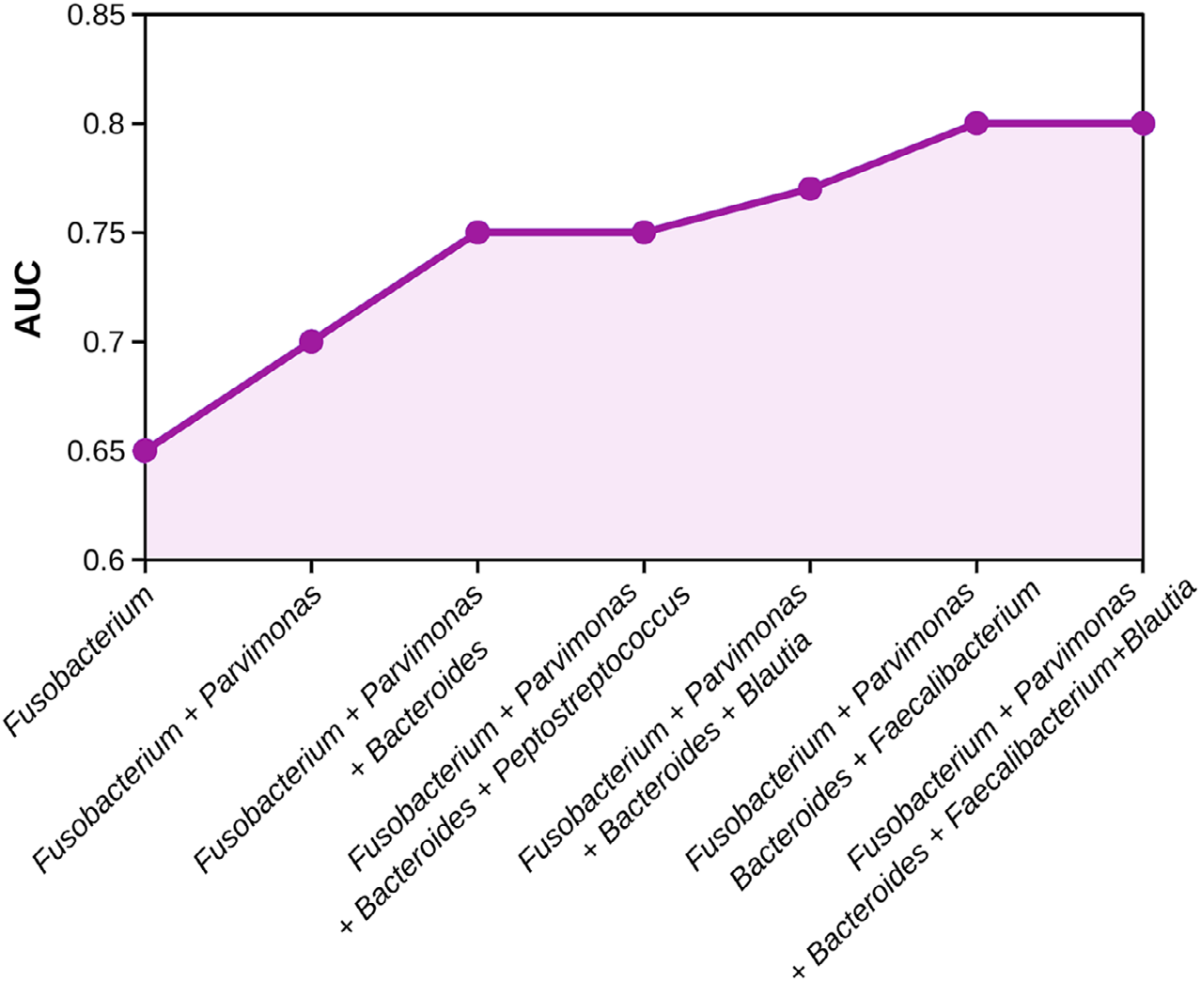
Prediction performance of bacterial biomarkers detected in fecal samples. Prediction performance is indicated as area under de curve (AUC) values obtained from receiver operating characteristic (ROC) curves of a leave‐one‐out cross validation method based on models with 1, 2, 3 or 4 bacterial genera.

The role of *Fusobacterium* as a potential biomarker in fecal samples was tested in our cohort. ROC curves were utilized to assess its discriminatory power, revealing an AUC of 0.65 when only *Fusobacterium* was included in the model (Fig. 11). Interestingly, the inclusion in the model of other over‐abundant bacteria in CRC feces like *Parvimonas* (oral) and *Bacteroides* (intestinal), improved the discriminatory power between CRC patients and non‐CRC individuals, obtaining an AUC of 0.75 (Fig. 11). However, the addition of other over‐abundant bacteria in CRC feces like *Peptostreptoccocus* did not increase the efficiency of the model, whereas the addition of healthy associated bacteria like *Blautia* or *Faecalibacterium* increased the AUC value up to 0.77 and 0.8, respectively. Feature selection using the Boruta algorithm confirmed the efficiency of *Fusobacterium* and *Parvimonas* as biomarkers for CRC. However, this algorithm suggested a combination of six other genera, namely, *Incertae sedis*, *Odoribacter*, *Faecalitalea*, UCG‐010, *Slackia* and *Parvimonas*, which achieved an AUC of 0.86, although the relative proportions of most of these poorly characterized bacteria were very low in the samples.

### 3.6 External validation

**Fig. 12:**
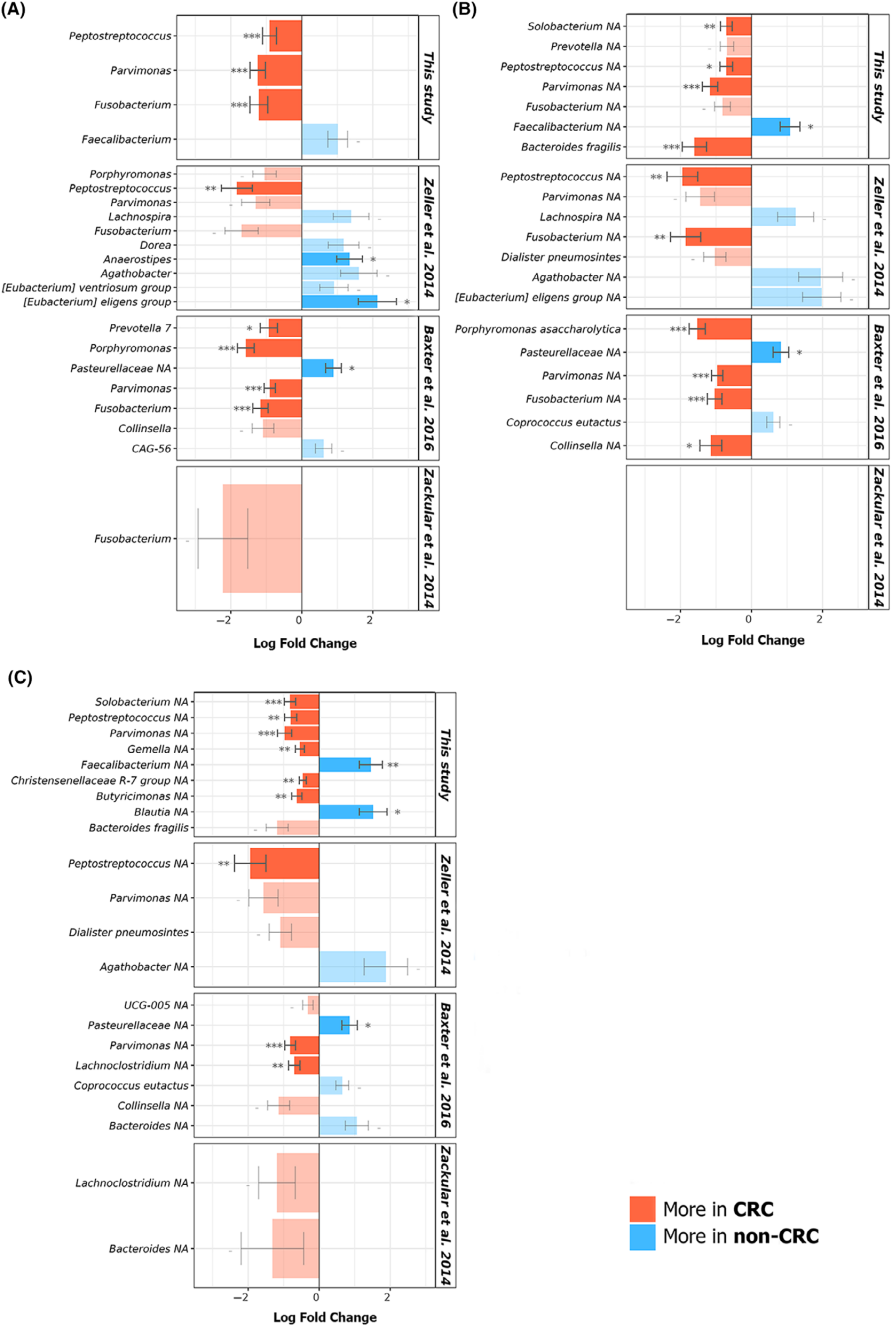
Differential abundance analysis (DAA) of the bacteriomes of fecal samples from colorectal cancer (CRC) patients and healthy individuals without any digestive disorders (non‐CRC) obtained from three different datasets compared to our study. Analyses were made at the (A) genus, (B) species and (C) amplicon sequencing variants (ASVs) levels. Effect size (log fold change), standard error and adjusted *P‐*values for each entry were obtained using the ANCOM‐BC method with a prevalence filter of 10% and subsequent Holm‐Bonferroni statistical test (*P‐*values ***: < 0.001; **: < 0.01; *: < 0.05).

When sequencing data obtained from those three previous studies (Table 1) were analyzed using our bioinformatic protocols, we have identified a notable enrichment of certain genera in stool samples from CRC patients (Fig. 12). These genera were *Peptostreptococcus*, *Prevotella*, *Porphyromonas*, *Parvimonas*, and *Fusobacterium*. Specifically, Zeller *et al*. [45] demonstrated that specific species such as *Bacteroides fragilis*, *Fusobacterium nucleatum*, *Parvimonas micra*, *Peptostreptococcus stomatis* and *Porphyromonas assaccharolytica* exhibit significantly higher abundance in fecal samples from cancer patients compared to those from healthy individuals.

## 4 Discussion

Current CRC follow‐up screening programs in Spain are based on FOBT tests, and if the result is positive, a colonoscopy is recommended [7]. Despite this, in many cases, the CRC symptoms are non‐specific, and the disease is only apparent when there is rectal bleeding or acute abdominal pain, which often corresponds to an advanced tumor stage, with a higher likelihood of distant metastasis [5, 8, 72]. At this critical phase, an individual's response to chemotherapy agents and their survival could also be compromised. Therefore, there is an urgent need for new, specific, and minimally invasive biomarkers to enhance the early detection of colorectal cancer.

Over the last few years, multiple studies have demonstrated that the gut microbiome of patients with CRC is significantly unbalanced. Colorectal dysbiosis is characterized by the loss of mutualistic species, significant reduction in microbial biodiversity, and strong decline in healthy microbiota functions [25, 29, 30, 35, 49, 73]. In the current study, we identified a clear over‐representation of common well‐known periodontal pathogens in all intestinal samples obtained from CRC diagnosed individuals. Interestingly, this finding suggests that some of these pathobionts could potentially serve as noninvasive fecal biomarkers for CRC diagnosis (specifically, *Fusobacterium* and *Parvimonas*). Most of the oral microorganisms detected in our samples were strict anaerobes (e.g., *Fusobacterium*, *Parvimonas*, *Prevotella*, *Peptostreptococcus*, and *Porphyromonas*), which are probably translocated from the subgingival cavity to the gut, as reported previously [34, 55, 74, 75]. As a matter of fact, our team demonstrated recently the translocation of *P. micra* from the gingival cavity to the gut in a patient diagnosed with CRC and advanced periodontal disease (PD) [55]. These oral bacteria, especially *Fusobacterium* and *Parvimonas*, were also over‐represented in samples from other CRC studied cohorts [23, 37, 49, 50, 76]. Therefore, we suggest that the co‐occurrence of these oral microorganisms in the gut, which are typical drivers of chronic oral inflammatory diseases, may play an important role in the development of colorectal tumors. The individual pro‐carcinogenic activities of some of these bacteria have been extensively studied over the last years [37, 77, 78, 79, 80, 81, 82, 83, 84]. Specifically, *Fusobacterium nucleatum* is a well‐known periodontal pathobiont and over the last 10 years its role in colorectal carcinogenesis was proven by different research groups [23, 24, 34, 36, 37, 51, 78, 85, 86, 87, 88, 89, 90, 91, 92, 93]. In this study, we found that the abundance of *Fusobacterium* increased from the normal mucosa to tumors, in agreement with previous studies [23, 36, 37, 88]. In addition, fecal samples from CRC patients showed to be significantly more enriched in *Fusobacterium* when compared to feces of non‐CRC individuals. Our findings agree with those of previous studies where *Fusobacterium* was also found to be over‐represented in neoplastic tissues and feces of bowel cancer patients [37, 51, 85, 87, 91]. In particular, this bacterium was positively associated with several factors linked with CRC development. *F. nucleatum* is related to CpG island methylator phenotype status, with microsatellite instability, somatic mutations [51, 91] and poorer patient survival prognosis [51, 88]. Additionally, it has been associated with chemoresistance [93], higher risk of CRC recurrence [90], and induction of metastases to distant tissues [92]. Furthermore, according to our results, another oral pathogen that overgrows in the colon of patients with CRC, is *Parvimonas*. This bacterium has also been associated with the development of CRC in previous studies [23, 31, 50, 76, 84]. Interestingly, the correlations between both gingival bacteria (*Parvimonas* and *Fusobacterium*) were previously associated with tumors belonging to the consensus molecular subtype 1 (CMS1) [25, 80]. Moreover, colonization of colorectal carcinomas by *Parvimonas* has been correlated with a decreased survival rate in patients with CRC. This phenomenon could be attributed to the ability of the bacterium to enhance tumorigenesis activity in the colon through epigenetic reprogramming of human intestinal cells and improvement of the Th17‐mediated immune response [80, 84].

In addition, *Peptostreptococcus* was another enriched genus in our CRC patient cohort, in agreement with different studies published before [94, 95]. *Peptostreptococcus anaerobius* promotes colonic cell proliferation and tumorigenesis by modulating the host immune environment [79]. It is interesting to note that both *Peptostreptococcus* and *Fusobacterium* adhere more efficiently to CRC cell lines than to normal colorectal lines [79, 89]. Concurrently, *Porphyromonas gingivalis*, another keystone pathogen of the red Socransky complex detected in CRC samples, is related to the proliferation of dysplastic lesions in the colon by activating the MAPK/ERK signaling molecular way [81], promoting the development of chronic inflammatory microenvironments in the gut [83]. Additionally, the differential abundance analysis supported previous studies where *B. fragilis* was proposed as a potential biomarker for carcinoma diagnosis due to its characteristic overgrowth in colorectal tumor tissues [76, 96].

Notably, it is crucial to underscore that the outcomes derived from analyzing external datasets [45, 70, 71] using our bioinformatics tools not only validate our findings but also substantiate the feasibility of three of our proposed bacterial biomarkers (*Fusobacterium*, *Parvimonas*, and *Bacteroides*) for CRC diagnosis. This observation suggests that these biomarkers may exhibit broad applicability, as evidenced by data collected from projects conducted in diverse regions, including France [45], the USA [70, 71], and Canada [71]. Moreover, a consistent depletion of *Faecalibacterium* and *Blautia* genera was observed in CRC individuals when compared to the non‐CRC group. Both genera are producers of short‐chain fatty acids (SCFAs) and have been described in previous studies as beneficial groups in the gut, exhibiting important immunemodulatory, anti‐inflammatory, and anti‐tumorigenic effects in host cells [97, 98, 99, 100]. These genera have also been found to exhibit reduced abundance in the colon of patients diagnosed with different bowel diseases, including cirrhosis and obesity, as detailed in multiple scientific reports [47, 101, 102, 103, 104]. Thus, the decrease in both taxonomic groups in the gut may be related to a possible proinflammatory status of the colorectal mucosa.

The strong connection observed in this study between the oral and gut microbiomes of patients with CRC has been reported in previous studies [30, 35, 46, 75, 105]. Similarly, our work supports that there is no clear overlap in microbiome composition between oral and fecal samples obtained from non‐CRC individuals. In Spain, a significant proportion of the adult population between the ages of 50 and 80 years, estimated at approximately 35%, is affected by PD [106]. In addition, recent studies revealed that patients diagnosed with PD have an increased risk of CRC development by ~ 44% when compared to individuals with good oral health, suggesting a positive correlation between oral disorders and CRC [36, 52]. Micro‐communities of various oral pathobionts, naturally found in saliva or gingival fluids [107], have the potential to migrate from the oral cavity to the gut [108, 109]. This migration can contribute to the colonization of new colorectal microenvironments, including abnormal gut structures, especially during the progression of the PD, when these pathobionts experience overgrowth [36, 52]. The similarities between the colorectal epithelium and the subgingival cavity, such as similar pH and low percentage of oxygen levels, create favorable conditions for the adaptation and overgrowth of these harmful periodontal organisms [110, 111]. In particular, the increased mucosal tissue mass, as occurs in dysplasia, provides an advantage for the growth of oral microbes due to the increased supply of nutrients. Usually, the formation of multi‐species consortia enhances the viability of individual bacteria, facilitates their adhesion and invasion of host cells, disrupts cell adhesive contacts, and further increases collective virulence as well as host vascular permeability [112, 113]. This could explain why inflammatory gum infections were triggered mostly by various microbes, suggesting that the microbial cluster detected in our study, could promote a strong proinflammatory effect in the gut, in a similar way that occurs in the oral cavity, altering eukaryotic cell signaling pathways [78, 79, 84, 92, 93]. However, it remains unknown how CRC‐associated bacteria interact in this dysbiotic environment. In this study, we observed that oral‐associated bacteria in tumors correlated with each other in a similar way as they did in the oral niche. Different bacterial complexes were established in the subgingival cavity by Socransky in the year 1998 [114]. Bacteria from orange and red complexes (proteolytic and strict anaerobes) were associated with PD whereas yellow and purple ones (facultative anaerobes and saccharolytic) were established as earlier colonizers and health associated. Nevertheless, it was shown that in supragingival liquid, members of both types of bacteria coexist in the biofilms [115] and bacteria from the “early colonizers” group (e.g., *Streptococcus*) protect strict anaerobes (e.g., *Fusobacterium*) from oxidative stress [116]. This synergism could explain the positive correlation among “early colonizers” and aerobes with strict anaerobes in the saliva fluid and normal colorectal tissues, observed in the current work. It is worth mentioning that although *Fusobacterium* and *Parvimonas* correlated positively in gingival and tissue samples, they did not correlate in saliva, suggesting that the synergy between these two pathogens is not as favorable or required in this oral environment. The lower correlation between strict and facultative anaerobic bacteria in carcinomas could indicate reduced oxygen irrigation in this microenvironment. In relation to this, Galeano *et al*. [111] conducted a study on the spatial distribution of intratumoral bacteria in CRC tumors. Their findings demonstrated that bacterial communities tend to populate microniches that are less vascularized and, therefore, have lower oxygen pressure. Although we were able to observe that the co‐occurrence patterns of oral bacteria in carcinomas were like those in the oral cavity, it is interesting to highlight that not all PD‐related pathogens can reach the gut environment. For example, although *Fusobacterium*, *Parvimonas*, and *Peptostreptoccocus* are present in adenocarcinomas, *Tannerella* and *Treponema* are absent in most patients. Furthermore, when we correlated the presence of bacteria in the saliva or subgingival fluid with their presence in colorectal tumors, no clear correlations were found. Therefore, our data indicate that the cluster of oral pathobionts detected in carcinomas represents a subset of the more complex ones present in the oral cavity. In addition, we observed that the genus *Hungatella* was highly correlated with the oral group, a taxonomic group already proposed as an efficient fecal CRC biomarker in combination with other oral microbes (*Parvimonas micra*, *Gemella morbillorum and Peptostreptococcus stomatis*) [105]. Although the specific mechanisms by which bacteria interact were not studied in our work, it was reported that *Hungatella* sp. were able to degrade glycosaminoglycans [117]. This capability may play an important role in facilitating the colonization of bacteria in the human gut epithelium, because glycosaminoglycans are important components of the gut mucosal layer. Another potential mechanism that could contribute to bacterial interactions in the gut is metabolic complementation. This concept involves different bacteria collaborating and sharing metabolic biosynthetic processes to perform different essential processes [118]. Concurrently, it is important to highlight that some strains of *B. fragilis*, which were also positively correlated with the cluster, can produce toxins (BFT; enterotoxigenic *Bacteroides fragilis*) and have been proposed as key inducers of CRC. The BFT is a zinc‐dependent metalloprotease that cleaves E‐cadherin (a cell–cell adhesion molecule), which increases colonic permeability and exposes the colon submucosa [119]. Thus, it is plausible that one of the relevant synergistic mechanisms between oral and intestinal bacteria is the facilitation of tissue colonization. Therefore, considering that: (a) *B. fragilis* can build polymicrobial, proinflammatory, and pro‐carcinogenic biofilms in the gut which can promote colorectal tumorogenesis [46] and (b) similar pathogenic communities have been found in the mucosa colorectal tissues and in the gingival fluid, we support the hypothesis that *B. fragilis* could have a relevant role during the development of the carcinoma biofilm [110]. Accordingly, these polymicrobial biofilms, whose composition changes throughout the carcinogenesis course [43, 120], may be the first step in creating an inflammatory state, enhancing the adenoma to carcinoma transition.

On the other hand, *Faecalibacterium* and *Agathobacter* are both health‐associated bacteria and butyrate‐producers [121, 122]. Butyrate, a SCFA, has anticarcinogenic effects [123] but it has been reported that the presence of *Fusobacerium* sp. had a negative effect on butyrate production during the CRC course [86]. In addition, our findings showed that both mucosal enterotypes identified in CRC patients in our study cohort had a similar likelihood of being colonized by oral bacteria. Moreover, recent research has shown that the *Prevotella‐*enriched enterotype may contribute to an increased occurrence of CRC [124], which aligns with the results obtained from our analysis, since the enterotype 2 was one of the most frequently detected in the intestinal samples of CRC patients.

Based on our bacteriome co‐occurrence data, we are convinced that the most effective approach for diagnosing CRC in fecal samples should not focus solely on the detection of one or two bacteria, but on a consortium of oral and intestinal pathogens. Therefore, we investigated the use of oral over‐abundant genera in the gut of patients with CRC as potential biomarkers in fecal samples. Initially, we tested *Fusobacterium* as a single diagnostic biomarker, obtaining a modest prediction performance (AUC 0.65). In a previous study, the authors reported similar results using *F. nucleatum* as a single diagnostic biomarker (AUC 0.68), enhancing the diagnostic accuracy by adding different clinical parameters into the model [125]. Specifically, in our work, we expanded this previous bacteriome biomarker model by integrating other over‐abundant bacteria in fecal samples from CRC patients (*Parvimonas* and *Bacteroides*) and another enriched genus in non‐CRC fecal samples (*Faecalibacterium*), resulting in an improved performance, up to an AUC of 0.8. The genera included in our model were: *Fusobacterium*, *Parvimonas*, *Bacteroides* and *Faecalibacterium*. Our analysis revealed that the combination of bacteria, including intestinal related genera (*Bacteroides* and *Faecalibacterium*), improves the discriminatory power between non‐CRC and CRC individuals. Moreover, it is important to remark that, most of these bacterial genera (*Fusobacterium*, *Parvimonas*, and *Bacteroides*) were also found to be over‐represented in adenomatous polyps, which are recognized as typical CRC precursor lesions [31, 38, 41]. Different studies have reported that bacteriome‐based biomarkers have the potential to distinguish individuals without gut neoplasia from those at high risk of developing CRC, even during the early stages of the disease, suggesting that gut microbiome dysbiosis appears before, during and after the adenoma to carcinoma transition [25, 35, 46, 47, 48, 49].

The data obtained in our study revealed that a specific consortium of oral and gut bacteria (*Fusobacterium*, *Parvimonas*, *Bacteroides* and *Faecalibacterium*) could be used as a predictive model for the detection of malignant neoplasms in the colon, even at early stages that are not detected by colonoscopy. Furthermore, an intriguing aspect of our study was that using the Boruta algorithm, we identified other taxa that could also be used as potential biomarkers for CRC. These included unidentified organisms found in low proportions as well as genera that have not been previously implicated in the carcinogenic process. More comprehensive cohorts in larger populations should be analyzed for validating these observations and confirm the additional predictive value provided by *Parvimonas* and other organisms. An extensive test of our present results will help us further assess the accuracy and reliability of this bacteriome‐based analysis. The observations obtained in this work highlight the complexity of the human microbiota and suggest the presence of novel microbial signatures and putative bacterial interactions that remain incompletely understood. In summary, our project emphasizes the importance of the ongoing exploration and comprehensive profiling of the human gut microbiome in the context of CRC.

In this study, we found that the fecal bacteriome composition of patients with CRC was significantly different from that of non‐CRC individuals. Moreover, the extensive analysis performed at both the oral and intestinal levels allowed us to assess a closer link between oral and gut microbes, determining an interesting consortium of microorganisms that could act as noninvasive CRC biomarkers. A major strength of our project is the use of high‐quality 16S rRNA metabarcoding data obtained from different samples from a total number of 93 CRC patients. Accordingly, bacteriome‐derived biomarkers have been proposed based on the study of different European patient cohorts [35, 38, 39, 40, 41, 42, 44, 45] but only one of these studies sampled the oral cavity of CRC patients [35]. In the present study, we profiled the oral and gut microbiomes of patients with CRC and compared their sequencing results with those obtained from the non‐CRC control group. Nevertheless, it is important to note that the non‐CRC and CRC groups were unbalanced in terms of sex, which could skew the results, as there is a higher risk of CRC development in men than in women (the 0–74 years old risk is ~ 1.83% in women and approximately double ~ 2.75%, in men [72]). We are aware that the control group consisted of a small number of non‐CRC individuals (*n* = 30) due to the limited number of companions and partners who agreed to donate F and S samples. Moreover, it is important to emphasize that, despite the small size of the non‐CRC cohort, these individuals shared similar characteristics such as age, weight, height, diet, and lifestyle as the CRC patients. These similarities make them suitable control groups for studies exploring health and disease microbiome dynamics.

In short, we believe that understanding the similarity between the colorectal epithelium and subgingival cavity can contribute to the construction of a new comprehensive understanding of dysbiosis and its impact on human health. Our data suggest that the bacterial oral consortium is the unit of colonization/infection of the colon tissue, as opposed to the role of individual species in the carcinogenic process. Therefore, further research is necessary to explore the mechanisms that allow the translocation of specific bacterial consortia to colorectal tumors, underlying the adaptation and growth of periodontal pathogens in the gut, and their potential implications in the initiation and progression of CRC. Moreover, studying the possible mechanisms of antagonism between microbes located in the colon could provide a promising new target for stopping gut dysbiosis and preventing inflammatory diseases and cancer.

## 5 Conclusions

This study provides new evidence that colorectal microbiota in patients diagnosed with CRC was enriched in several oral pathobionts, compared to samples from non‐CRC individuals, in which these pathogens were practically absent or only traces of one or two oral genera are detected. We believe that oral microbes translocate to the gut probably by forming clusters and afterwards they colonize the colorectal mucosa, being easily detected in the stool of patients. The periodontal bacterial associations reported in the present work may enhance the development of a proinflammatory microenvironment, collaborating with the onset of tumorigenesis. To conclude, we propose that the cluster formed by *Fusobacterium*, *Parvimonas*, *Bacteroides* and *Faecalibacterium* could be used as an excellent biomarker for diagnosis of CRC. However, a better understanding of the role of the oral‐gut microbiome axis in pathogenesis will be advantageous for the precise diagnosis and prognosis of CCR, and for effective treatments. We also aimed to highlight that PD is a risk factor for CRC initiation. Therefore, oral treatments could contribute to reducing the incidence and prevalence of cancer and CRC screening for patients diagnosed with PD may be useful to improve the early diagnosis of CRC.

## Conflict of interest

The authors declare no conflict of interest.

## Author contributions

JAV, MP and AM conceived and designed the study. MP, JAV, AM and GB supervised this study. KC‐P, JAV and MP obtained F and S samples from both CRC and non‐CRC groups. MP managed the informed consents from all individuals. JFN obtained fresh colorectal tissue samples from surgery. ÁC, LSE and BO‐A performed the anatomopathological analyses of tumor samples from CRC patients. KC‐P, NT‐T, MN‐A, JAV, SR‐F and PN processed the samples and performed all sequencing procedures. PA‐M, KC‐P, JAV, EM‐DA and SL performed bioinformatics analysis related to DNA sequencing. EB, MC‐D and AM performed correlation, enterotype and biomarker bioinformatic analyses. KC‐P, PA‐M and EB designed and created figures and tables. SP‐L obtained subgingival samples and made dental check‐ups for patients with CRC. IG‐R, NM‐L and LMAA (Deceased) followed up on the oncological patients. KC‐P, MP, AM, JAV, EB and MC‐D wrote the manuscript. All authors reviewed and approved the publication of this manuscript.

### Peer review

The peer review history for this article is available at https://www.webofscience.com/api/gateway/wos/peer‐review/10.1002/1878‐0261.13604.

## Data Availability

The raw sequence reads generated from 16S rRNA gene sequencing analyses by MiSeq (Illumina) are available in the NCBI SRA database under the accession code PRJNA911189. The detailed scripts and documentation are available at GitHub (https://github.com/Pablo‐Aja‐Macaya/CRC‐16S‐study).
